# Supplementary figures and images for: Hierarchical modelling of immunoglobulin coated bacteria in dogs with chronic enteropathy shows reduction in coating with disease remission but marked inter-individual and treatment-response variability
Source: PLoS One. 2021 Aug 19;16(8):e0255012. doi: 10.1371/journal.pone.0255012 (PMC8376084; doi:10.1371/journal.pone.0255012)

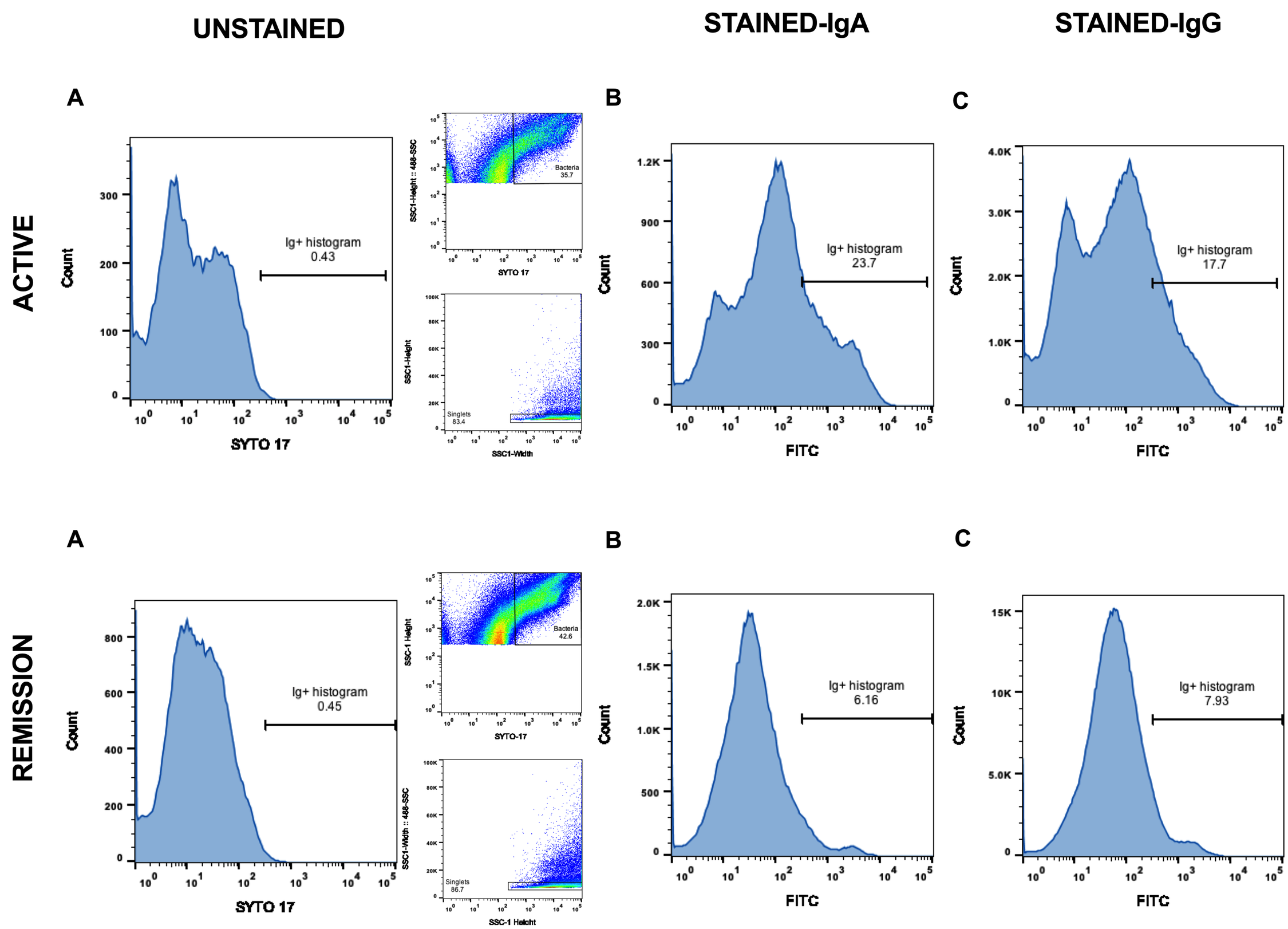

Supplement: S1 Fig — For microbial fraction identification the trigger was set up based on side scatter properties. Then, singlets were selected based on SSC-width vs SCC-height. FITC-negative window population was set up in samples containing only SYTO 17 (A). These windows were applied to samples stained with FITC-IgA (B) or FITC-IgG (C) to distinguish the negative population from the positive one. (TIF) [file pone.0255012.s001.tif]

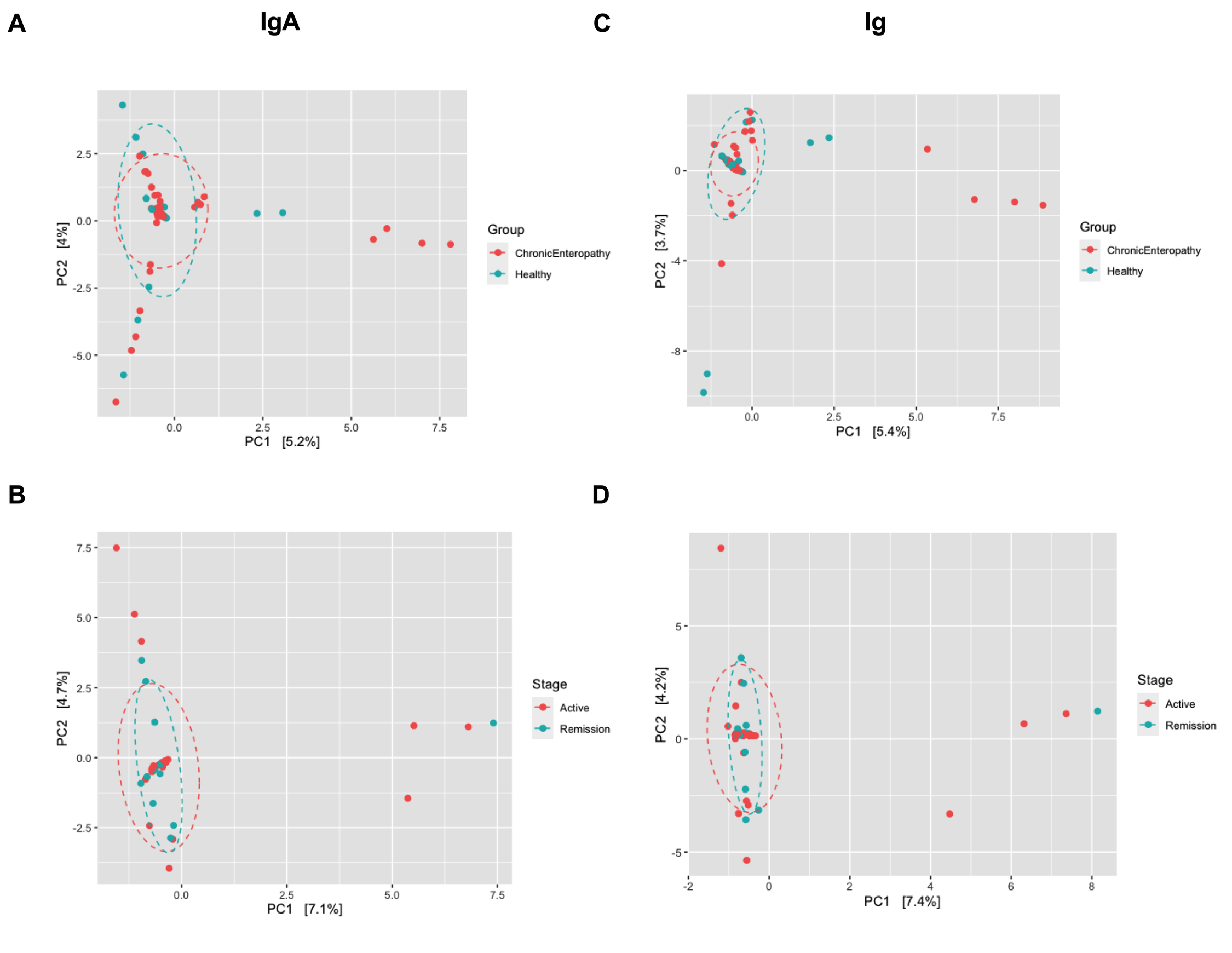

Supplement: S2 Fig — (A) Principal coordinate analysis (PCA) plot IgA healthy versus CE (B) PCA plot IgG healthy versus CE (C) PCA plot IgA stage CE dogs. (D) PCA plot IgG stage CE dogs. (TIF) [file pone.0255012.s002.tif]

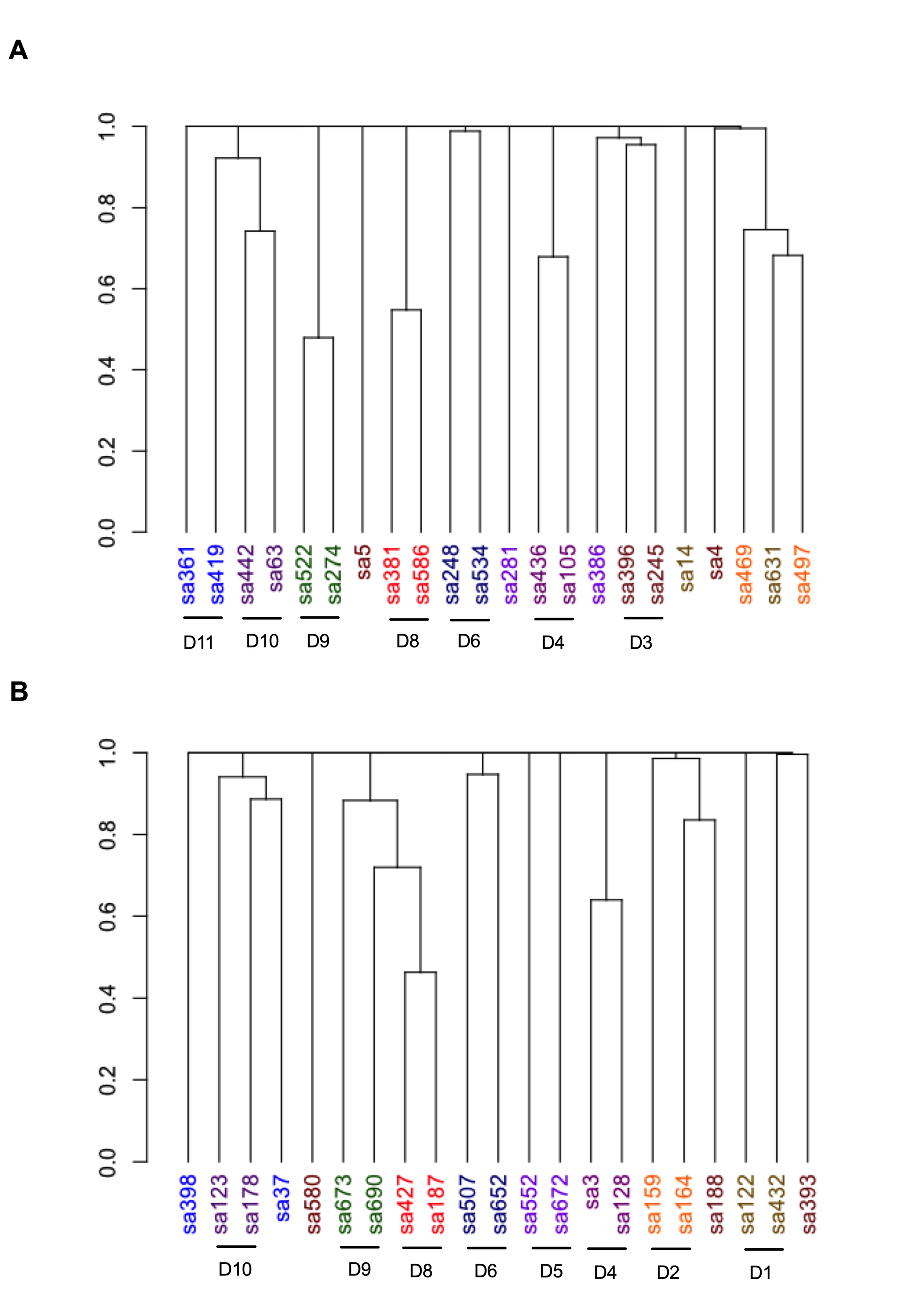

Supplement: S3 Fig — (A) IgA and (B) IgG populations. Colours represent different dogs. (TIF) [file pone.0255012.s003.tif]

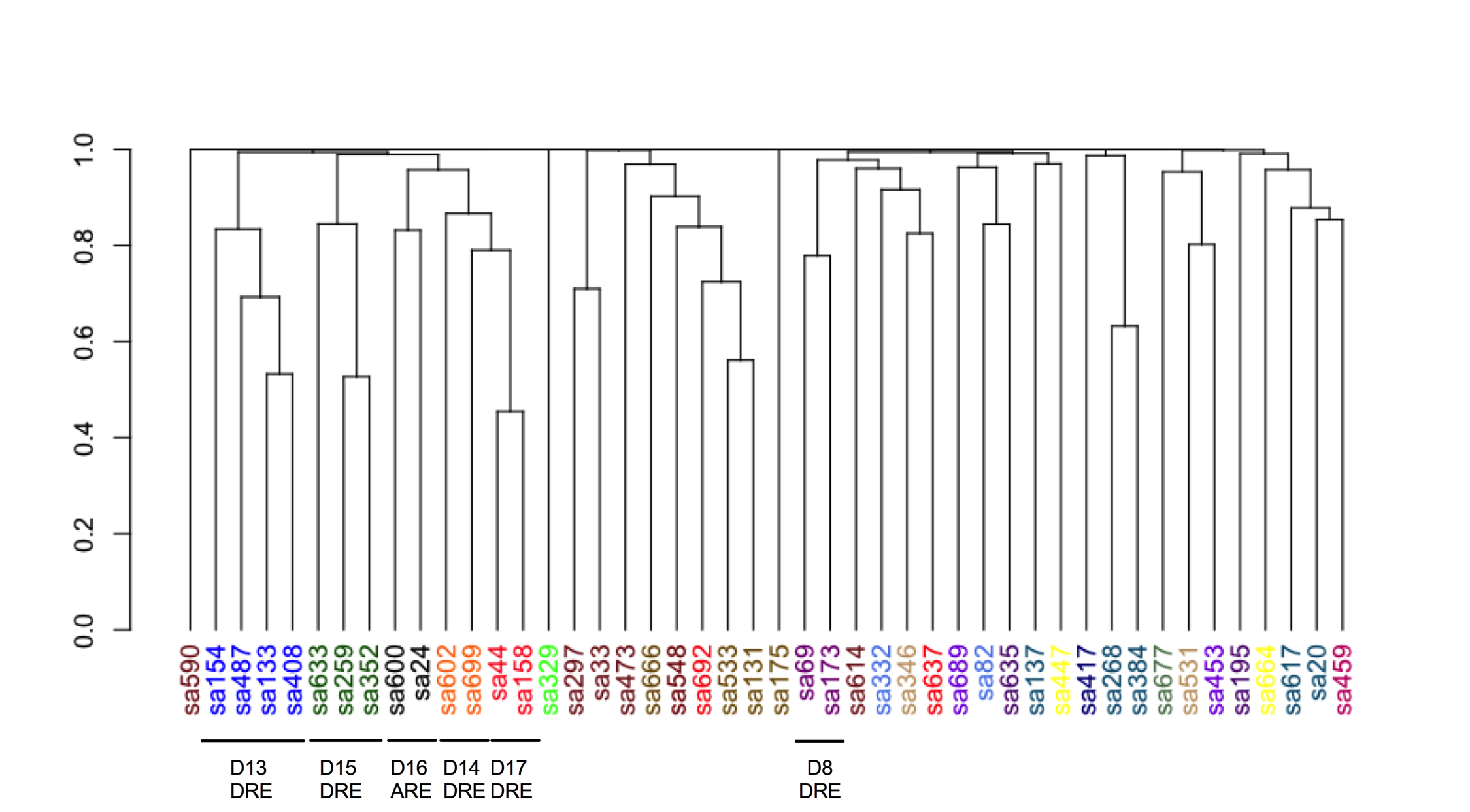

Supplement: S4 Fig — Colours represent different dogs. (TIF) [file pone.0255012.s004.tif]

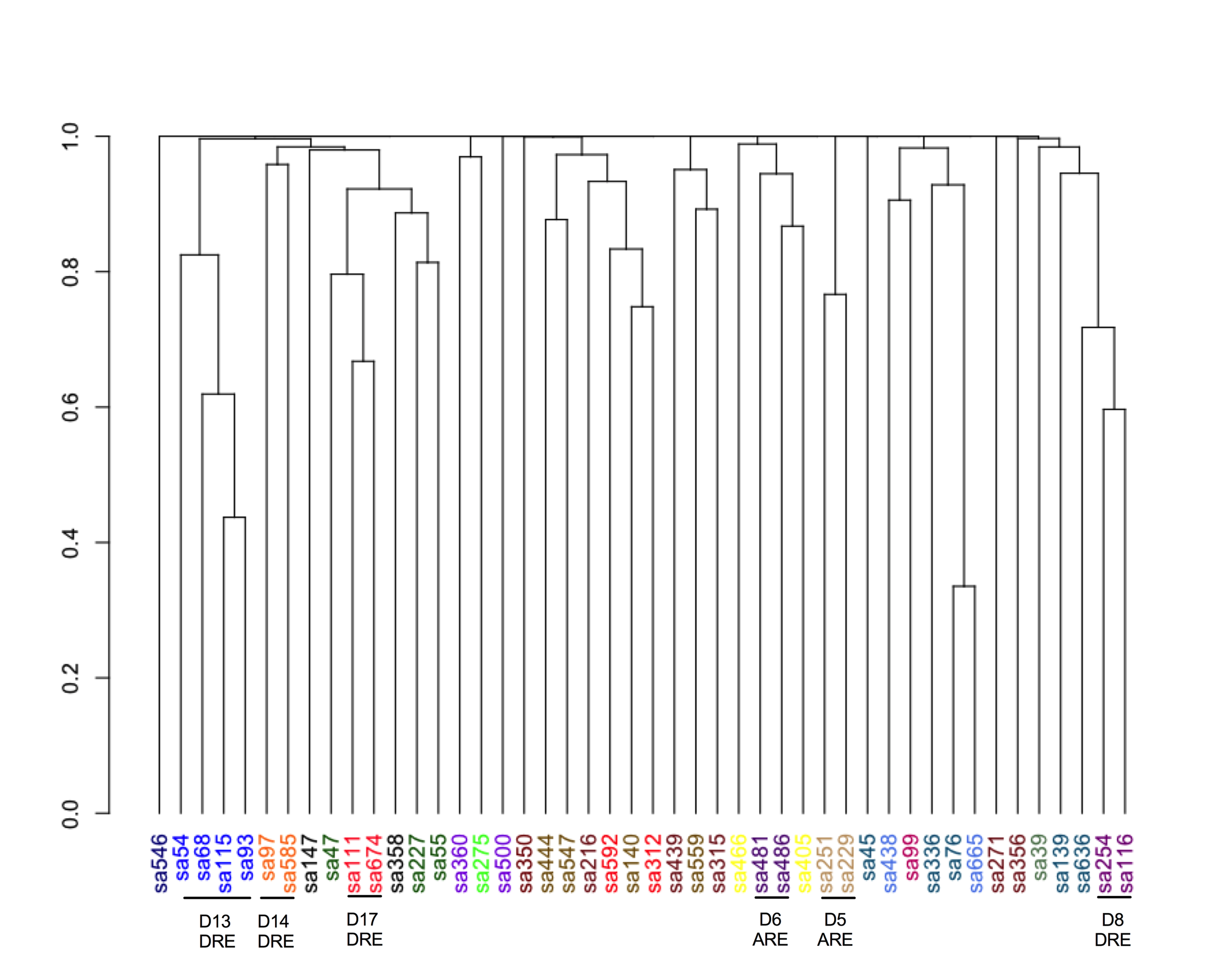

Supplement: S5 Fig — Colours represent different dogs. (TIF) [file pone.0255012.s005.tif]

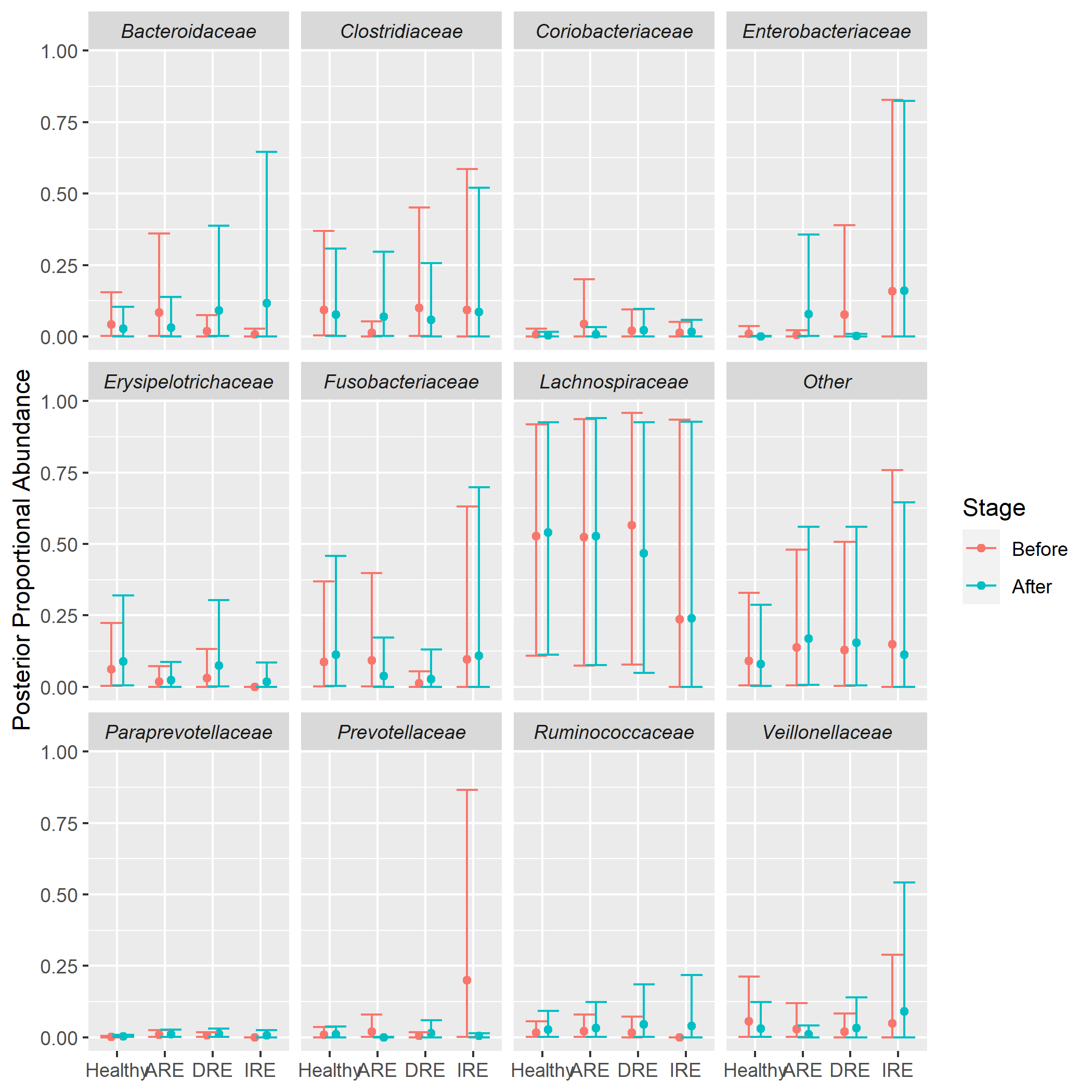

Supplement: S6 Fig — DRE: Diet-responsive enteropathy. ARE: Antibiotic-responsive enteropathy. IRE: Immunosuppressant-responsive enteropathy. Before corresponds to V1 in healthy dogs and active disease in CE dogs. After corresponds to V2 in healthy dogs and remission in CE dogs. Top eleven of the most representative families. Other includes the rest of the families. The central credible interval corresponds to 50% and the outer interval to 90%. (TIF) [file pone.0255012.s006.tif]
