# Supplementary material for: Hierarchical modelling of immunoglobulin coated bacteria in dogs with chronic enteropathy shows reduction in coating with disease remission but marked inter-individual and treatment-response variability
Source: PLoS One. 2021 Aug 19;16(8):e0255012. doi: 10.1371/journal.pone.0255012 (PMC8376084; doi:10.1371/journal.pone.0255012)
Supplement: S1 Table — (DOCX) [file pone.0255012.s007.docx]

| **S1 Table. Metadata information of dogs with chronic enteropathy** | | | | | | | | | | | | | |
| --- | --- | --- | --- | --- | --- | --- | --- | --- | --- | --- | --- | --- | --- |
| **ID** | **Breed** | **Age (Y)** | **Neutering status** | **Classification****CE** | **Localisation** | | **CCECAI** **Active** | **CCECAI****Remission** | **Duration****(months)** | **Concurrent diseases** | **Treatment** | **Study period** | **#****Visits** |
| CE1 | Spoodle | 5 | Male castrated | IRE | Mixed | 5 | | 0 | 24 | PolyarthritisBronchopathy | Z/D Hills®Sensitivity Royal canin®Hypoallergenic Royal canin®PrednisoloneClorambucil(Gabapentin) | Phase 2 | 5 |
| CE2 | JapaneseSpitz | 1.5 | Female spayed | ARE | Mixed | 8 | | 2 | 4 | None | Sensitivity Royal canin®Hypoallergenic Royal canin®Oxytetracycline | Phase 1 | 2 |
| CE3 | Whippet | 4 | Female spayed | ARE | SI | 6 | | NA | 15 | Separation anxiety | Sensitivity Royal canin®Hypoallergenic Royal canin®Oxytetracycline | Phase 2 | 1  Active |
| CE4 | Border Collie | 5 | Male castrated | ARE | SI | Unknown | | Unknown | 14 | EPI | Hypoallergenic Royal canin®Oxytetracycline(Creon®) | Phase 2 | 2 |
| CE5 | Maltese Cross | 6.5 | Male castrated | ARE | SI | 11 | | 2 | 31 | None | Sensitivity Royal canin®Oxytetracycline | Phase 1 | 2 |
| CE6 | Labrador Retriever | 2 | Female spayed | ARE | SI | 7 | | 1 | 5 | Incontinence (USMI) | Sensitivity Royal canin®Oxytetracycline(stilboestrol) | Phase 1 | 2 |
| CE7 | Golder Retriever | 5 | Male castrated | DRE | SI | 7 | | 1 | 18 | None | Hypoallergenic Royal canin® | Phase 2 | 4 |
| CE8 | Staffordshire Bull Terrier | 2 | Male castrated | DRE | Mixed | 11 | | 2 | 24 | None | Hypoallergenic Royal canin® | Phase 1 | 2 |
| CE9 | Labrador Retriever | 10 | Female spayed | IRE | Mixed | 7 | | 0 | 14 | PLE EPI | Hypoallergenic Royal canin®OxytetracyclinePrednisoloneClorambucil(Creon®) | Phase 1 | 1  Remission |
| CE 10 | Labrador Retriever | 10 | Female spayed | DRE | Mixed | 7 | | 4 | 12 | Otitis | Z/D Hills® | Phase 1 | 2 |
| CE 11 | Great Dane | 2,5 | Male castrated | DRE | LI | 4 | | 0 | 28 | None | Sensitivity Royal canin® | Phase 1 | 2 |
| CE 12 | Maltese Terrier | 3,5 | Female entire | DRE | Unknown | Unknown | | Unknown | 4 | None | Z/D Hills®Hypoallergenic Royal canin®Omeprazole | Phase 1 | 1 |
| CE 13 | GSD | 14 | Female spayed | DRE | Mixed | 6 | | 0 | 7 | None | Z/D Hills®Sensitivity Royal canin® Hypoallergenic Royal canin® | Phase 2 | 3 |
| CE 14 | GSD | 1 | Male entire | DRE | Mixed | 3 | | 0 | 2 | None | Sensitivity Royal canin® | Phase 1 | 2 |
| CE 15 | Chihuahua | 9 | Male castrated | DRE | Mixed | Unknown | | Unknown | 24 | None | Z/D Hills®Sensitivity Royal canin®Hypoallergenic Royal canin® | Phase 2 | 3 |
| CE 16 | GSD | 1.5 | Female spayed | ARE | Mixed | 12 | | 1 | 3 | Anxiety | Sensitivity Royal canin®Hypoallergenic Royal canin®Oxytetracycline(Fluoxitine, diazepam) | Phase 1 | 2 |
| CE 17 | Maltese Cross | 5 | Female Spayed | DRE | SI | 6 | | 0 | 12 | None | Sensitivity Royal canin®Hypoallergenic Royal canin® | Phase 2 | 1  Active |
| CE 18 | Greyhound | 2 | Female spayed | ARE | Mixed | 5 | | 0 | Unknown | Greyhound alopecia | Hypoallergenic Royal canin®Oxytetracycline | Phase 2 | 5 |
| CE 19 | Basset Hound | 3 | Male entire | ARE | Mixed | 5 | | 0 | 24 | None | Sensitivity Royal canin®Hypoallergenic Royal canin®Oxytetracycline | Phase 1 | 2 |
| CE 20 | Flat CoatedRetriever | 9 | Male castrated | IRE | SI | Unknown | | Unknown | 8 | Trigeminal neuropathy | Z/D Hills®Hypoallergenic Royal canin®OxytetracyclinePrednisolone | Phase 2 | 1  Active |
| CE 21 | Toy Poodle | 2.5 | Male castrated | DRE | LI | 9 | | 0 | 18 | None | Sensitivity Royal canin®Oxytetracycline | Phase 1 | 2 |
| CE 22 | Weimaraner | 1.7 | Male castrated | DRE | Mixed | 4 | | 0 | 3 | None | Hypoallergenic Royal canin® | Phase 1 | 2 |

GSD: German Shepherd dog. DRE: Diet-responsive enteropathy. ARE: Antibiotic-responsive enteropathy. IRE: Immunosuppressant-responsive enteropathy. SI: Small Intestine. LI: Large intestine. CCECAI: canine chronic enteropathy activity index. EPI: Exocrine pancreatic insufficiency. USMI: Urethral Sphincter Mechanism Incompetence. [PLE: Protein-losing enteropathy. () Treatment not associated with CE. Y: years.](https://www.ncbi.nlm.nih.gov/pubmed/29131672)
