# Supplementary material for: Hierarchical modelling of immunoglobulin coated bacteria in dogs with chronic enteropathy shows reduction in coating with disease remission but marked inter-individual and treatment-response variability
Source: PLoS One. 2021 Aug 19;16(8):e0255012. doi: 10.1371/journal.pone.0255012 (PMC8376084; doi:10.1371/journal.pone.0255012)
Supplement: S2 Table — (DOCX) [file pone.0255012.s008.docx]

**S2 Table. Metadata information of Healthy dogs**

| ID | Breed | Age  (Y) | Sex/neutering status | BCS | Diet | Treats | Coprophagia/  pica | Worming | Level of exercise | Travel history | Other pets at home |
| --- | --- | --- | --- | --- | --- | --- | --- | --- | --- | --- | --- |
| H1 | Labrador Retriever | 2 | Male/neutered | 4/9 | J/d hills® | Pig ears, biscuits, cooked meat. 1 per day | NO | Drontal® | High | National | 1dog, 2 cats,1 guinea pig |
| H2 | Leonberger | 7 | Female/spayed | 4/9 | Black hawk chicken rice® | Pig ears, raw chicken frames, 1 once per week | NO | Drontal® | Moderate | New Zealand | 1 dog |
| H3 | German wirehaired pointer | 6 | Male/entire | 4/9 | Advance dry food® | Biscuits, raw meat, chicken necks, sardines  Amount varies according to activity | Cow manure, kangaroo | Interceptor® | Moderate | National | 2 dogs |
| H4 | Nova Scotia Duck Tolling Retriever | 3 | Female/spayed | 5/9 | T/d Hills® | Pig ears  1/once per week | NO | Sentinel® | Intense | None | None |
| H5 | Cairn Terrier | 5 | Female/spayed | 7/9 | T/d Hills® | Carrot, broccoli  Occasionally | NO | Sentinel® | Moderate | None | None |
| H6 | Siberian husky cross | 11 | Male/neutered | 5/9 | J/d Hills® | Homemade food, biscuits  1/ once per day | Dog, cat and possum faeces | Drontal® | Mild | Canada | None |
| H7 | Australian cattle dog cross | 8 | Male/neutered | 4/9 | J/d Hills® | Pig ears, biscuits, cooked meat  1 per day | NO | Drontal® | Moderate | National | 1 dog, 2 cats, 1 guinea pig |
| H8 | Australian kelpie | 4 | Female/spayed | 5/9 | J/d Hills® | Meatballs, liver treats  1/three times per week | NO | Canex® | Intense | None | 2 cats |
| H9 | Australian kelpie | 9 | Female/spayed | 4/9 | Royal canin® | Dental chews, cow/sheep bones  1/ three times per week | NO | Comfortis® | Moderate | National | 1 cat, reptiles |
| H10 | German wirehaired pointer | 1 | Female/entire | 4/9 | Advance dry food® | Biscuits, raw meat, chicken necks, sardines  Amount varies according to activity | NO | Interceptor® | Moderate | National | 2 dogs |
| H11 | Terrier cross | 5 | Female/spayed | 3/9 | Hills adult light® | Pig ears, biscuit  1/twice per week | NO | Drontal® | Intense | Europe | 2 cats |

Y: year
