# Supplementary material for: Hierarchical modelling of immunoglobulin coated bacteria in dogs with chronic enteropathy shows reduction in coating with disease remission but marked inter-individual and treatment-response variability
Source: PLoS One. 2021 Aug 19;16(8):e0255012. doi: 10.1371/journal.pone.0255012 (PMC8376084; doi:10.1371/journal.pone.0255012)
Supplement: S3 Table — (DOCX) [file pone.0255012.s009.docx]

**S3 Table. Estimate proportions of sorted coated bacteria and credible intervals.**

| **Immunoglobulin** | **Group** | **Classification CE** | **Stage** | **Estimate** | **Q5** | **Q95** |
| --- | --- | --- | --- | --- | --- | --- |
| A | Healthy | NA | Before | 28.3 | 20.6 | 36.9 |
| A | Healthy | NA | After | 25.3 | 18.3 | 33.4 |
| A | CE | DRE | Before | 39.9 | 29.9 | 50.7 |
| A | CE | DRE | After | 29.7 | 21.2 | 39.3 |
| A | CE | ARE | Before | 35.1 | 24.9 | 46.1 |
| A | CE | ARE | After | 28.1 | 19.2 | 38.2 |
| A | CE | IRE | Before | 63.7 | 45.2 | 79.8 |
| A | CE | IRE | After | 26.7 | 13.6 | 43 |
| G | Healthy | NA | Before | 27.8 | 21.5 | 34.9 |
| G | Healthy | NA | After | 23.8 | 18.2 | 30.3 |
| G | CE | DRE | Before | 29.2 | 22.3 | 37 |
| G | CE | DRE | After | 21.5 | 16 | 28 |
| G | CE | ARE | Before | 29.2 | 22.3 | 37 |
| G | CE | ARE | After | 18.2 | 12.9 | 24.5 |
| G | CE | IRE | Before | 39.1 | 24.9 | 54.1 |
| G | CE | IRE | After | 22.5 | 12.7 | 34.1 |

CE: Chronic enteropathy. DRE: Diet-responsive enteropathy. ARE: Antibiotic-responsive enteropathy. IRE: Immunosuppressant-responsive enteropathy. ‘Before’ corresponds to V1 in healthy dogs and active disease in CE dogs. ‘After’ corresponds to V2 in healthy dogs and remission in CE dogs.
