# Supplementary material for: Hierarchical modelling of immunoglobulin coated bacteria in dogs with chronic enteropathy shows reduction in coating with disease remission but marked inter-individual and treatment-response variability
Source: PLoS One. 2021 Aug 19;16(8):e0255012. doi: 10.1371/journal.pone.0255012 (PMC8376084; doi:10.1371/journal.pone.0255012)
Supplement: S4 Table — (DOCX) [file pone.0255012.s010.docx]

**S4 Table. Predictive values and confidence intervals of Shannon diversity**

| **Immunoglobulin** | | **Group** | **Classification**  **CE** | **Stage** | **Response** | **SE** | **Lower CL** | **Upper**  **CL** |
| --- | --- | --- | --- | --- | --- | --- | --- | --- |
| Input | Healthy | | NA | Before | 2.58 | 0.168 | 2.26 | 2.94 |
| Input | Healthy | | NA | After | 2.82 | 0.181 | 2.48 | 3.21 |
| Input | CE | | DRE | Before | 2.7 | 0.18 | 2.35 | 3.09 |
| Input | CE | | DRE | After | 3.17 | 0.234 | 2.73 | 3.67 |
| Input | CE | | ARE | Before | 3.07 | 0.229 | 2.64 | 3.56 |
| Input | CE | | ARE | After | 2.86 | 0.226 | 2.45 | 3.35 |
| Input | CE | | IRE | Before | 2.78 | 0.345 | 2.16 | 3.56 |
| Input | CE | | IRE | After | 3.31 | 0.464 | 2.5 | 4.38 |
| A | Healthy | | NA | Before | 1.87 | 0.148 | 1.6 | 2.19 |
| A | Healthy | | NA | After | 2.29 | 0.172 | 1.98 | 2.66 |
| A | CE | | DRE | Before | 2.32 | 0.176 | 1.99 | 2.7 |
| A | CE | | DRE | After | 2.45 | 0.216 | 2.06 | 2.92 |
| A | CE | | ARE | Before | 2.66 | 0.229 | 2.24 | 3.16 |
| A | CE | | ARE | After | 2.05 | 0.192 | 1.7 | 2.47 |
| A | CE | | IRE | Before | 2.09 | 0.296 | 1.58 | 2.77 |
| A | CE | | IRE | After | 2.49 | 0.422 | 1.78 | 3.48 |
| G | Healthy | | NA | Before | 2.13 | 0.162 | 1.83 | 2.47 |
| G | Healthy | | NA | After | 2.05 | 0.161 | 1.75 | 2.39 |
| G | CE | | DRE | Before | 2.41 | 0.183 | 2.07 | 2.8 |
| G | CE | | DRE | After | 2.41 | 0.212 | 2.03 | 2.87 |
| G | CE | | ARE | Before | 2.57 | 0.221 | 2.16 | 3.05 |
| G | CE | | ARE | After | 2.03 | 0.191 | 1.68 | 2.45 |
| G | CE | | IRE | Before | 2.16 | 0.306 | 1.63 | 2.87 |
| G | CE | | IRE | After | 2.99 | 0.508 | 2.14 | 4.19 |

CE: Chronic enteropathy. DRE: Diet-responsive enteropathy. ARE: Antibiotic-responsive enteropathy. IRE: Immunosuppressant-responsive enteropathy. ‘Before’ corresponds to V1 in healthy dogs and Active disease in CE dogs. ‘After’ corresponds to V2 in healthy dogs and Remission in CE dogs. Input (Pre-sort). CL: Confidence limit.
