# Supplementary material for: Hierarchical modelling of immunoglobulin coated bacteria in dogs with chronic enteropathy shows reduction in coating with disease remission but marked inter-individual and treatment-response variability
Source: PLoS One. 2021 Aug 19;16(8):e0255012. doi: 10.1371/journal.pone.0255012 (PMC8376084; doi:10.1371/journal.pone.0255012)
Supplement: S5 Table — (DOCX) [file pone.0255012.s011.docx]

**S5 Table. The subject estimate values of the proportional abundance of the different families in the input (pre-sort) population and their credible intervals.**

| **Ig** | **Stage** | **Classification_CE** | **Taxon** | **Estimate** | **Est.Error** | **Q5** | **Q95** |
| --- | --- | --- | --- | --- | --- | --- | --- |
| \| Presort \| \| --- \| \| Presort \| \| Presort \| \| Presort \| \| Presort \| \| Presort \| \| Presort \| \| Presort \| \| Presort \| \| Presort \| \| Presort \| \| Presort \| \| Presort \| \| Presort \| \| Presort \| \| Presort \| \| Presort \| \| Presort \| \| Presort \| \| Presort \| \| Presort \| \| Presort \| \| Presort \| \| Presort \| \| Presort \| \| Presort \| \| Presort \| \| Presort \| \| Presort \| \| Presort \| \| Presort \| \| Presort \| \| Presort \| \| Presort \| \| Presort \| \| Presort \| \| Presort \| \| Presort \| \| Presort \| \| Presort \| \| Presort \| \| Presort \| \| Presort \| \| Presort \| \| Presort \| \| Presort \| \| Presort \| \| Presort \| \| Presort \| \| Presort \| \| Presort \| \| Presort \| \| Presort \| \| Presort \| \| Presort \| \| Presort \| \| Presort \| \| Presort \| \| Presort \| \| Presort \| \| Presort \| \| Presort \| \| Presort \| \| Presort \| \| Presort \| \| Presort \| \| Presort \| \| Presort \| \| Presort \| \| Presort \| \| Presort \| \| Presort \| \| Presort \| \| Presort \| \| Presort \| \| Presort \| \| Presort \| \| Presort \| \| Presort \| \| Presort \| \| Presort \| \| Presort \| \| Presort \| \| Presort \| \| Presort \| \| Presort \| \| Presort \| \| Presort \| \| Presort \| \| Presort \| \| Presort \| \| Presort \| \| Presort \| \| Presort \| \| Presort \| \| Presort \| | \| Before \| \| --- \| \| Before \| \| Before \| \| Before \| \| After \| \| After \| \| After \| \| After \| \| Before \| \| Before \| \| Before \| \| Before \| \| After \| \| After \| \| After \| \| After \| \| Before \| \| Before \| \| Before \| \| Before \| \| After \| \| After \| \| After \| \| After \| \| Before \| \| Before \| \| Before \| \| Before \| \| After \| \| After \| \| After \| \| After \| \| Before \| \| Before \| \| Before \| \| Before \| \| After \| \| After \| \| After \| \| After \| \| Before \| \| Before \| \| Before \| \| Before \| \| After \| \| After \| \| After \| \| After \| \| Before \| \| Before \| \| Before \| \| Before \| \| After \| \| After \| \| After \| \| After \| \| Before \| \| Before \| \| Before \| \| Before \| \| After \| \| After \| \| After \| \| After \| \| Before \| \| Before \| \| Before \| \| Before \| \| After \| \| After \| \| After \| \| After \| \| Before \| \| Before \| \| Before \| \| Before \| \| After \| \| After \| \| After \| \| After \| \| Before \| \| Before \| \| Before \| \| Before \| \| After \| \| After \| \| After \| \| After \| \| Before \| \| Before \| \| Before \| \| Before \| \| After \| \| After \| \| After \| \| After \| | \| DRE \| \| --- \| \| ARE \| \| IRE \| \| Healthy \| \| DRE \| \| ARE \| \| IRE \| \| Healthy \| \| DRE \| \| ARE \| \| IRE \| \| Healthy \| \| DRE \| \| ARE \| \| IRE \| \| Healthy \| \| DRE \| \| ARE \| \| IRE \| \| Healthy \| \| DRE \| \| ARE \| \| IRE \| \| Healthy \| \| DRE \| \| ARE \| \| IRE \| \| Healthy \| \| DRE \| \| ARE \| \| IRE \| \| Healthy \| \| DRE \| \| ARE \| \| IRE \| \| Healthy \| \| DRE \| \| ARE \| \| IRE \| \| Healthy \| \| DRE \| \| ARE \| \| IRE \| \| Healthy \| \| DRE \| \| ARE \| \| IRE \| \| Healthy \| \| DRE \| \| ARE \| \| IRE \| \| Healthy \| \| DRE \| \| ARE \| \| IRE \| \| Healthy \| \| DRE \| \| ARE \| \| IRE \| \| Healthy \| \| DRE \| \| ARE \| \| IRE \| \| Healthy \| \| DRE \| \| ARE \| \| IRE \| \| Healthy \| \| DRE \| \| ARE \| \| IRE \| \| Healthy \| \| DRE \| \| ARE \| \| IRE \| \| Healthy \| \| DRE \| \| ARE \| \| IRE \| \| Healthy \| \| DRE \| \| ARE \| \| IRE \| \| Healthy \| \| DRE \| \| ARE \| \| IRE \| \| Healthy \| \| DRE \| \| ARE \| \| IRE \| \| Healthy \| \| DRE \| \| ARE \| \| IRE \| \| Healthy \| | \| *Paraprevotellaceae* \| \| --- \| \| *Paraprevotellaceae* \| \| *Paraprevotellaceae* \| \| *Paraprevotellaceae* \| \| *Paraprevotellaceae* \| \| *Paraprevotellaceae* \| \| *Paraprevotellaceae* \| \| *Paraprevotellaceae* \| \| *Bacteroidaceae* \| \| *Bacteroidaceae* \| \| *Bacteroidaceae* \| \| *Bacteroidaceae* \| \| *Bacteroidaceae* \| \| *Bacteroidaceae* \| \| *Bacteroidaceae* \| \| *Bacteroidaceae* \| \| *Clostridiaceae* \| \| *Clostridiaceae* \| \| *Clostridiaceae* \| \| *Clostridiaceae* \| \| *Clostridiaceae* \| \| *Clostridiaceae* \| \| *Clostridiaceae* \| \| *Clostridiaceae* \| \| *Coriobacteriaceae* \| \| *Coriobacteriaceae* \| \| *Coriobacteriaceae* \| \| *Coriobacteriaceae* \| \| *Coriobacteriaceae* \| \| *Coriobacteriaceae* \| \| *Coriobacteriaceae* \| \| *Coriobacteriaceae* \| \| *Enterobacteriaceae* \| \| *Enterobacteriaceae* \| \| *Enterobacteriaceae* \| \| *Enterobacteriaceae* \| \| *Enterobacteriaceae* \| \| *Enterobacteriaceae* \| \| *Enterobacteriaceae* \| \| *Enterobacteriaceae* \| \| *Erysipelotrichaceae* \| \| *Erysipelotrichaceae* \| \| *Erysipelotrichaceae* \| \| *Erysipelotrichaceae* \| \| *Erysipelotrichaceae* \| \| *Erysipelotrichaceae* \| \| *Erysipelotrichaceae* \| \| *Erysipelotrichaceae* \| \| *Fusobacteriaceae* \| \| *Fusobacteriaceae* \| \| *Fusobacteriaceae* \| \| *Fusobacteriaceae* \| \| *Fusobacteriaceae* \| \| *Fusobacteriaceae* \| \| *Fusobacteriaceae* \| \| *Fusobacteriaceae* \| \| *Lachnospiraceae* \| \| *Lachnospiraceae* \| \| *Lachnospiraceae* \| \| *Lachnospiraceae* \| \| *Lachnospiraceae* \| \| *Lachnospiraceae* \| \| *Lachnospiraceae* \| \| *Lachnospiraceae* \| \| *Prevotellaceae* \| \| *Prevotellaceae* \| \| *Prevotellaceae* \| \| *Prevotellaceae* \| \| *Prevotellaceae* \| \| *Prevotellaceae* \| \| *Prevotellaceae* \| \| *Prevotellaceae* \| \| *Ruminococcaceae* \| \| *Ruminococcaceae* \| \| *Ruminococcaceae* \| \| *Ruminococcaceae* \| \| *Ruminococcaceae* \| \| *Ruminococcaceae* \| \| *Ruminococcaceae* \| \| *Ruminococcaceae* \| \| *Veillonellaceae* \| \| *Veillonellaceae* \| \| *Veillonellaceae* \| \| *Veillonellaceae* \| \| *Veillonellaceae* \| \| *Veillonellaceae* \| \| *Veillonellaceae* \| \| *Veillonellaceae* \| \| Other \| \| Other \| \| Other \| \| Other \| \| Other \| \| Other \| \| Other \| \| Other \| | \| 0.006123994 \| \| --- \| \| 0.009552305 \| \| 1.04E-05 \| \| 0.001833081 \| \| 0.011492139 \| \| 0.010362203 \| \| 0.006977758 \| \| 0.00375631 \| \| 0.018199516 \| \| 0.084547256 \| \| 0.007921411 \| \| 0.042129818 \| \| 0.091331825 \| \| 0.031858162 \| \| 0.115826723 \| \| 0.028019805 \| \| 0.099303639 \| \| 0.012893747 \| \| 0.092753212 \| \| 0.092473098 \| \| 0.058084089 \| \| 0.068968598 \| \| 0.085158568 \| \| 0.076176429 \| \| 0.020727188 \| \| 0.044547504 \| \| 0.013131449 \| \| 0.006836775 \| \| 0.021540559 \| \| 0.008205968 \| \| 0.016314751 \| \| 0.004237592 \| \| 0.076164714 \| \| 0.005138193 \| \| 0.157884029 \| \| 0.008759121 \| \| 0.002492186 \| \| 0.07881505 \| \| 0.16052616 \| \| 0.000651648 \| \| 0.031151901 \| \| 0.018466943 \| \| 1.57E-05 \| \| 0.06161946 \| \| 0.073856721 \| \| 0.022605535 \| \| 0.018066246 \| \| 0.08819544 \| \| 0.012324504 \| \| 0.091925666 \| \| 0.095442276 \| \| 0.086997979 \| \| 0.027670532 \| \| 0.03886212 \| \| 0.109469533 \| \| 0.112393693 \| \| 0.565130381 \| \| 0.523623547 \| \| 0.235609783 \| \| 0.526649272 \| \| 0.466569302 \| \| 0.527969665 \| \| 0.240544825 \| \| 0.539359142 \| \| 0.004580765 \| \| 0.020112629 \| \| 0.199780605 \| \| 0.00943873 \| \| 0.014342608 \| \| 0.000344646 \| \| 0.004288786 \| \| 0.010094578 \| \| 0.016836348 \| \| 0.021229358 \| \| 1.53E-05 \| \| 0.016522545 \| \| 0.04519261 \| \| 0.032596404 \| \| 0.03945688 \| \| 0.026644616 \| \| 0.019561969 \| \| 0.029299467 \| \| 0.049161463 \| \| 0.055673076 \| \| 0.032230272 \| \| 0.010707304 \| \| 0.090089753 \| \| 0.031274777 \| \| 0.12989508 \| \| 0.138663386 \| \| 0.148274307 \| \| 0.091067046 \| \| 0.155197159 \| \| 0.168704345 \| \| 0.113280018 \| \| 0.079195971 \| | \| 0.00588892 \| \| --- \| \| 0.00748731 \| \| 2.04E-05 \| \| 0.00129717 \| \| 0.00949412 \| \| 0.00823293 \| \| 0.01026285 \| \| 0.00272074 \| \| 0.04924903 \| \| 0.12475854 \| \| 0.04335571 \| \| 0.0651932 \| \| 0.14193905 \| \| 0.06318242 \| \| 0.21033539 \| \| 0.04799076 \| \| 0.15479326 \| \| 0.03408696 \| \| 0.19515134 \| \| 0.12868094 \| \| 0.10844498 \| \| 0.11143311 \| \| 0.18414863 \| \| 0.11381602 \| \| 0.05756287 \| \| 0.08392612 \| \| 0.06282371 \| \| 0.01884215 \| \| 0.05801903 \| \| 0.02286982 \| \| 0.07541865 \| \| 0.01244841 \| \| 0.14108067 \| \| 0.01484884 \| \| 0.25756622 \| \| 0.02342196 \| \| 0.01000939 \| \| 0.12890557 \| \| 0.25781297 \| \| 0.0022349 \| \| 0.06211272 \| \| 0.0362611 \| \| 0.00066633 \| \| 0.08286136 \| \| 0.11192237 \| \| 0.04420991 \| \| 0.06681955 \| \| 0.10892707 \| \| 0.03744738 \| \| 0.14154366 \| \| 0.20292094 \| \| 0.13241137 \| \| 0.06670226 \| \| 0.08108706 \| \| 0.21762175 \| \| 0.15553737 \| \| 0.28625713 \| \| 0.27521131 \| \| 0.30643951 \| \| 0.25676134 \| \| 0.28484699 \| \| 0.27517578 \| \| 0.30457868 \| \| 0.25761737 \| \| 0.01324707 \| \| 0.04142894 \| \| 0.2787681 \| \| 0.01657067 \| \| 0.03097471 \| \| 0.0012728 \| \| 0.02950682 \| \| 0.01760797 \| \| 0.03683236 \| \| 0.03841036 \| \| 0.0001188 \| \| 0.02614027 \| \| 0.0758704 \| \| 0.05406076 \| \| 0.10763227 \| \| 0.03964062 \| \| 0.04435008 \| \| 0.05704023 \| \| 0.12931357 \| \| 0.08313767 \| \| 0.06342977 \| \| 0.02454834 \| \| 0.18346082 \| \| 0.05412341 \| \| 0.16927303 \| \| 0.15815447 \| \| 0.23893551 \| \| 0.11269213 \| \| 0.18229918 \| \| 0.1791272 \| \| 0.20543788 \| \| 0.10229146 \| | \| 0.00064033 \| \| --- \| \| 0.00145427 \| \| 1.60E-07 \| \| 0.00033694 \| \| 0.0015842 \| \| 0.00157291 \| \| 0.00021285 \| \| 0.00066045 \| \| 0.00018745 \| \| 0.00245328 \| \| 1.53E-06 \| \| 0.00156549 \| \| 0.00181163 \| \| 0.00070169 \| \| 0.00011484 \| \| 0.00095474 \| \| 0.00137674 \| \| 0.00022246 \| \| 4.07E-05 \| \| 0.00318078 \| \| 0.00077878 \| \| 0.00159065 \| \| 3.66E-05 \| \| 0.00237884 \| \| 0.00012299 \| \| 0.00073362 \| \| 1.27E-06 \| \| 0.00012674 \| \| 0.00015251 \| \| 0.00010599 \| \| 1.87E-06 \| \| 7.27E-05 \| \| 0.00058797 \| \| 4.25E-05 \| \| 9.52E-05 \| \| 0.00015536 \| \| 1.21E-05 \| \| 0.00100972 \| \| 0.00011042 \| \| 1.03E-05 \| \| 0.00057775 \| \| 0.00053568 \| \| 4.46E-12 \| \| 0.00307026 \| \| 0.00198395 \| \| 0.00065548 \| \| 9.85E-06 \| \| 0.00455883 \| \| 5.77E-05 \| \| 0.00154085 \| \| 2.77E-05 \| \| 0.00193617 \| \| 0.00016446 \| \| 0.00048675 \| \| 4.47E-05 \| \| 0.00274834 \| \| 0.07757625 \| \| 0.07391075 \| \| 0.00047052 \| \| 0.10822945 \| \| 0.0492864 \| \| 0.07584965 \| \| 0.00062433 \| \| 0.11284343 \| \| 5.92E-05 \| \| 0.00051368 \| \| 0.00051135 \| \| 0.00038287 \| \| 0.0002419 \| \| 7.22E-06 \| \| 1.49E-06 \| \| 0.0004003 \| \| 0.0002986 \| \| 0.00075342 \| \| 2.02E-09 \| \| 0.00089474 \| \| 0.00112356 \| \| 0.00126217 \| \| 4.01E-05 \| \| 0.0014474 \| \| 0.00021534 \| \| 0.0007218 \| \| 2.66E-05 \| \| 0.00228858 \| \| 0.00046217 \| \| 0.00023557 \| \| 7.91E-05 \| \| 0.00116691 \| \| 0.00325603 \| \| 0.00585483 \| \| 0.0002338 \| \| 0.00470915 \| \| 0.00510996 \| \| 0.0079102 \| \| 0.00015459 \| \| 0.00382238 \| | \| 0.0173418 \| \| --- \| \| 0.02468037 \| \| 4.27E-05 \| \| 0.00441317 \| \| 0.02972457 \| \| 0.02658351 \| \| 0.02511711 \| \| 0.00914288 \| \| 0.07381025 \| \| 0.3601088 \| \| 0.02809715 \| \| 0.15520853 \| \| 0.38668821 \| \| 0.13822545 \| \| 0.64548084 \| \| 0.10342747 \| \| 0.45104915 \| \| 0.05214293 \| \| 0.58646333 \| \| 0.36992215 \| \| 0.25700532 \| \| 0.29597582 \| \| 0.51983307 \| \| 0.30792272 \| \| 0.09486434 \| \| 0.20014582 \| \| 0.05065182 \| \| 0.02776771 \| \| 0.09614307 \| \| 0.03297175 \| \| 0.05856992 \| \| 0.01697644 \| \| 0.38867373 \| \| 0.02101088 \| \| 0.82860665 \| \| 0.03594955 \| \| 0.00969734 \| \| 0.35740851 \| \| 0.82429835 \| \| 0.00251793 \| \| 0.13182466 \| \| 0.07327404 \| \| 6.51E-06 \| \| 0.22352241 \| \| 0.30375033 \| \| 0.08747502 \| \| 0.08615542 \| \| 0.31974711 \| \| 0.05426045 \| \| 0.39842871 \| \| 0.6305857 \| \| 0.36958852 \| \| 0.13045937 \| \| 0.17334786 \| \| 0.69844452 \| \| 0.45790915 \| \| 0.95844843 \| \| 0.93661096 \| \| 0.93584097 \| \| 0.91870463 \| \| 0.92654472 \| \| 0.94066142 \| \| 0.92713026 \| \| 0.92636147 \| \| 0.01747308 \| \| 0.07967196 \| \| 0.86622883 \| \| 0.0353001 \| \| 0.05897167 \| \| 0.00130651 \| \| 0.01333029 \| \| 0.03710918 \| \| 0.07168755 \| \| 0.08019382 \| \| 4.67E-05 \| \| 0.05674605 \| \| 0.18595308 \| \| 0.1237827 \| \| 0.21731857 \| \| 0.09204374 \| \| 0.08314037 \| \| 0.11995287 \| \| 0.28846714 \| \| 0.21331621 \| \| 0.14043365 \| \| 0.04134515 \| \| 0.54179356 \| \| 0.12290486 \| \| 0.5066157 \| \| 0.47985771 \| \| 0.75756312 \| \| 0.32849217 \| \| 0.5601645 \| \| 0.55985606 \| \| 0.64567055 \| \| 0.28775381 \| |

DRE: Diet-responsive enteropathy. ARE: Antibiotic-responsive enteropathy. IRE: Immunosuppressant-responsive enteropathy. ‘Before’ corresponds to V1 in healthy dogs and active disease in CE dogs. ‘After’ corresponds to V2 in healthy dogs and remission in CE dogs. Top eleven of the most representative families. Other includes the rest of the families.
