# Supplementary material for: Hierarchical modelling of immunoglobulin coated bacteria in dogs with chronic enteropathy shows reduction in coating with disease remission but marked inter-individual and treatment-response variability
Source: PLoS One. 2021 Aug 19;16(8):e0255012. doi: 10.1371/journal.pone.0255012 (PMC8376084; doi:10.1371/journal.pone.0255012)
Supplement: S6 Table — (DOCX) [file pone.0255012.s012.docx]

**S6 Table. Estimates of the immunoglobulin A ratios and their credible intervals.**

| **Disease** |  | **Stage** | **Taxon** | **Q5** | **Q25** | **Q50** | **Q75** | **Q95** |
| --- | --- | --- | --- | --- | --- | --- | --- | --- |
| \| IRE \| \| --- \| \| IRE \| \| DRE \| \| DRE \| \| ARE \| \| ARE \| \| Healthy \| \| Healthy \| \| IRE \| \| IRE \| \| DRE \| \| DRE \| \| ARE \| \| ARE \| \| Healthy \| \| Healthy \| \| IRE \| \| IRE \| \| DRE \| \| DRE \| \| ARE \| \| ARE \| \| Healthy \| \| Healthy \| \| IRE \| \| IRE \| \| DRE \| \| DRE \| \| ARE \| \| ARE \| \| Healthy \| \| Healthy \| \| IRE \| \| IRE \| \| DRE \| \| DRE \| \| ARE \| \| ARE \| \| Healthy \| \| Healthy \| \| IRE \| \| IRE \| \| DRE \| \| DRE \| \| ARE \| \| ARE \| \| Healthy \| \| Healthy \| \| IRE \| \| IRE \| \| DRE \| \| DRE \| \| ARE \| \| ARE \| \| Healthy \| \| Healthy \| \| IRE \| \| IRE \| \| DRE \| \| DRE \| \| ARE \| \| ARE \| \| Healthy \| \| Healthy \| \| IRE \| \| IRE \| \| DRE \| \| DRE \| \| ARE \| \| ARE \| \| Healthy \| \| Healthy \| \| IRE \| \| IRE \| \| DRE \| \| DRE \| \| ARE \| \| ARE \| \| Healthy \| \| Healthy \| \| IRE \| \| IRE \| \| DRE \| \| DRE \| \| ARE \| \| ARE \| \| Healthy \| \| Healthy \| \| IRE \| \| IRE \| \| DRE \| \| DRE \| \| ARE \| \| ARE \| \| Healthy \| \| Healthy \| |  | \| After \| \| --- \| \| Before \| \| After \| \| Before \| \| After \| \| Before \| \| After \| \| Before \| \| After \| \| Before \| \| After \| \| Before \| \| After \| \| Before \| \| After \| \| Before \| \| After \| \| Before \| \| After \| \| Before \| \| After \| \| Before \| \| After \| \| Before \| \| After \| \| Before \| \| After \| \| Before \| \| After \| \| Before \| \| After \| \| Before \| \| After \| \| Before \| \| After \| \| Before \| \| After \| \| Before \| \| After \| \| Before \| \| After \| \| Before \| \| After \| \| Before \| \| After \| \| Before \| \| After \| \| Before \| \| After \| \| Before \| \| After \| \| Before \| \| After \| \| Before \| \| After \| \| Before \| \| After \| \| Before \| \| After \| \| Before \| \| After \| \| Before \| \| After \| \| Before \| \| After \| \| Before \| \| After \| \| Before \| \| After \| \| Before \| \| After \| \| Before \| \| After \| \| Before \| \| After \| \| Before \| \| After \| \| Before \| \| After \| \| Before \| \| After \| \| Before \| \| After \| \| Before \| \| After \| \| Before \| \| After \| \| Before \| \| After \| \| Before \| \| After \| \| Before \| \| After \| \| Before \| \| After \| \| Before \| | \| *Bacteroidaceae* \| \| --- \| \| *Bacteroidaceae* \| \| *Bacteroidaceae* \| \| *Bacteroidaceae* \| \| *Bacteroidaceae* \| \| *Bacteroidaceae* \| \| *Bacteroidaceae* \| \| *Bacteroidaceae* \| \| *Clostridiaceae* \| \| *Clostridiaceae* \| \| *Clostridiaceae* \| \| *Clostridiaceae* \| \| *Clostridiaceae* \| \| *Clostridiaceae* \| \| *Clostridiaceae* \| \| *Clostridiaceae* \| \| *Coriobacteriaceae* \| \| *Coriobacteriaceae* \| \| *Coriobacteriaceae* \| \| *Coriobacteriaceae* \| \| *Coriobacteriaceae* \| \| *Coriobacteriaceae* \| \| *Coriobacteriaceae* \| \| *Coriobacteriaceae* \| \| *Enterobacteriaceae* \| \| *Enterobacteriaceae* \| \| *Enterobacteriaceae* \| \| *Enterobacteriaceae* \| \| *Enterobacteriaceae* \| \| *Enterobacteriaceae* \| \| *Enterobacteriaceae* \| \| *Enterobacteriaceae* \| \| *Erysipelotrichaceae* \| \| *Erysipelotrichaceae* \| \| *Erysipelotrichaceae* \| \| *Erysipelotrichaceae* \| \| *Erysipelotrichaceae* \| \| *Erysipelotrichaceae* \| \| *Erysipelotrichaceae* \| \| *Erysipelotrichaceae* \| \| *Fusobacteriaceae* \| \| *Fusobacteriaceae* \| \| *Fusobacteriaceae* \| \| *Fusobacteriaceae* \| \| *Fusobacteriaceae* \| \| *Fusobacteriaceae* \| \| *Fusobacteriaceae* \| \| *Fusobacteriaceae* \| \| *Lachnospiraceae* \| \| *Lachnospiraceae* \| \| *Lachnospiraceae* \| \| *Lachnospiraceae* \| \| *Lachnospiraceae* \| \| *Lachnospiraceae* \| \| *Lachnospiraceae* \| \| *Lachnospiraceae* \| \| Other \| \| Other \| \| Other \| \| Other \| \| Other \| \| Other \| \| Other \| \| Other \| \| *Paraprevotellaceae* \| \| *Paraprevotellaceae* \| \| *Paraprevotellaceae* \| \| *Paraprevotellaceae* \| \| *Paraprevotellaceae* \| \| *Paraprevotellaceae* \| \| *Paraprevotellaceae* \| \| *Paraprevotellaceae* \| \| *Prevotellaceae* \| \| *Prevotellaceae* \| \| *Prevotellaceae* \| \| *Prevotellaceae* \| \| *Prevotellaceae* \| \| *Prevotellaceae* \| \| *Prevotellaceae* \| \| *Prevotellaceae* \| \| *Ruminococcaceae* \| \| *Ruminococcaceae* \| \| *Ruminococcaceae* \| \| *Ruminococcaceae* \| \| *Ruminococcaceae* \| \| *Ruminococcaceae* \| \| *Ruminococcaceae* \| \| *Ruminococcaceae* \| \| *Veillonellaceae* \| \| *Veillonellaceae* \| \| *Veillonellaceae* \| \| *Veillonellaceae* \| \| *Veillonellaceae* \| \| *Veillonellaceae* \| \| *Veillonellaceae* \| \| *Veillonellaceae* \| | \| 0.02733068 \| \| --- \| \| 1.31934488 \| \| 0.03551566 \| \| 0.30897624 \| \| 0.00705767 \| \| 0.03589979 \| \| 0.09119551 \| \| 0.11921206 \| \| 0.20031211 \| \| 0.11030017 \| \| 0.88561603 \| \| 0.38459548 \| \| 0.21819204 \| \| 0.35383435 \| \| 0.22496675 \| \| 0.18961337 \| \| 5.06714641 \| \| 0.49186476 \| \| 0.29944638 \| \| 0.39957833 \| \| 0.3541405 \| \| 1.88219745 \| \| 0.78161858 \| \| 0.2143551 \| \| 0.68699916 \| \| 0.36577697 \| \| 0.01552314 \| \| 0.23885877 \| \| 0.20279574 \| \| 0.69903684 \| \| 1.1710783 \| \| 0.256521 \| \| 3.03540425 \| \| 0.01017005 \| \| 0.07462666 \| \| 1.07117513 \| \| 0.13126472 \| \| 2.17770512 \| \| 0.53062066 \| \| 0.42489053 \| \| 0.19094582 \| \| 0.69861796 \| \| 0.11208938 \| \| 0.20711524 \| \| 0.02596843 \| \| 0.20831992 \| \| 0.13713586 \| \| 0.15387012 \| \| 0.31848325 \| \| 0.81656773 \| \| 1.04878234 \| \| 1.017341 \| \| 1.04682459 \| \| 0.95223224 \| \| 0.91304181 \| \| 1.05666274 \| \| 0.65386938 \| \| 1.12757149 \| \| 0.62207949 \| \| 0.59961165 \| \| 0.18526264 \| \| 0.79561672 \| \| 1.864777 \| \| 0.79810789 \| \| 0.00098982 \| \| 6.42E-05 \| \| 0.0804607 \| \| 0.29116189 \| \| 0.27721412 \| \| 0.06348564 \| \| 0.65998888 \| \| 0.71252075 \| \| 1.04E-07 \| \| 0.07001656 \| \| 0.00765448 \| \| 0.04587607 \| \| 0.00012588 \| \| 0.0367946 \| \| 0.10436963 \| \| 0.07017521 \| \| 0.67417338 \| \| 240.151305 \| \| 0.59211089 \| \| 1.47398336 \| \| 0.91639354 \| \| 0.75930316 \| \| 0.4797801 \| \| 0.72708498 \| \| 0.21570389 \| \| 0.26401967 \| \| 0.13602687 \| \| 0.35793472 \| \| 0.01887321 \| \| 0.03699899 \| \| 0.0178805 \| \| 0.01114369 \| | \| 0.04673347 \| \| --- \| \| 1.74120024 \| \| 0.04122954 \| \| 0.33247246 \| \| 0.00844659 \| \| 0.04005598 \| \| 0.10545502 \| \| 0.13587654 \| \| 0.34205405 \| \| 0.14620521 \| \| 1.02972539 \| \| 0.41495785 \| \| 0.25369968 \| \| 0.39191907 \| \| 0.25982622 \| \| 0.21649662 \| \| 9.08036712 \| \| 0.71248203 \| \| 0.34828692 \| \| 0.43376498 \| \| 0.4254118 \| \| 2.08353443 \| \| 0.90988808 \| \| 0.24724933 \| \| 1.17358347 \| \| 0.47971539 \| \| 0.02197208 \| \| 0.25635896 \| \| 0.23596668 \| \| 0.77221521 \| \| 1.3500962 \| \| 0.2939262 \| \| 5.24795237 \| \| 0.6607942 \| \| 0.08685561 \| \| 1.15779669 \| \| 0.15326305 \| \| 2.40630608 \| \| 0.61255478 \| \| 0.48638366 \| \| 0.3256557 \| \| 0.9197752 \| \| 0.13009005 \| \| 0.22281589 \| \| 0.03071871 \| \| 0.23083665 \| \| 0.15908087 \| \| 0.17576595 \| \| 0.5436491 \| \| 1.07665714 \| \| 1.21566661 \| \| 1.0890736 \| \| 1.21531277 \| \| 1.05287341 \| \| 1.05347044 \| \| 1.20652861 \| \| 1.12452038 \| \| 1.48922195 \| \| 0.72011404 \| \| 0.64375083 \| \| 0.21612734 \| \| 0.88116978 \| \| 2.1550078 \| \| 0.91218701 \| \| 0.00246663 \| \| 0.0003853 \| \| 0.09326471 \| \| 0.3122442 \| \| 0.32205257 \| \| 0.07072937 \| \| 0.76698308 \| \| 0.81532236 \| \| 2.40E-06 \| \| 0.1069865 \| \| 0.00893872 \| \| 0.04922435 \| \| 0.00037759 \| \| 0.04107894 \| \| 0.12071095 \| \| 0.08025068 \| \| 1.16678456 \| \| 644.141568 \| \| 0.68936691 \| \| 1.58283062 \| \| 1.06791549 \| \| 0.84259708 \| \| 0.55633763 \| \| 0.83148152 \| \| 0.37180129 \| \| 0.34839346 \| \| 0.15798764 \| \| 0.38465625 \| \| 0.02413773 \| \| 0.04255714 \| \| 0.02091444 \| \| 0.01289314 \| | \| 0.06978936 \| \| --- \| \| 2.38535006 \| \| 0.05037677 \| \| 0.37607503 \| \| 0.01086579 \| \| 0.04442812 \| \| 0.11905077 \| \| 0.16306354 \| \| 0.50890137 \| \| 0.20159617 \| \| 1.25498004 \| \| 0.46938024 \| \| 0.32531939 \| \| 0.43432325 \| \| 0.29239848 \| \| 0.26056277 \| \| 13.3880669 \| \| 0.98950541 \| \| 0.42555539 \| \| 0.49038812 \| \| 0.54327636 \| \| 2.31233563 \| \| 1.02829731 \| \| 0.2990692 \| \| 1.75323924 \| \| 0.66370647 \| \| 0.02893298 \| \| 0.28985052 \| \| 0.30443213 \| \| 0.85459948 \| \| 1.51830618 \| \| 0.3531139 \| \| 7.77080545 \| \| 11.9052137 \| \| 0.10597669 \| \| 1.3061016 \| \| 0.19734992 \| \| 2.67028055 \| \| 0.68674544 \| \| 0.58264216 \| \| 0.48470522 \| \| 1.27093458 \| \| 0.15896035 \| \| 0.25269554 \| \| 0.03910645 \| \| 0.25547221 \| \| 0.17874825 \| \| 0.21077603 \| \| 0.81169588 \| \| 1.4847902 \| \| 1.48495685 \| \| 1.23138121 \| \| 1.56028024 \| \| 1.165378 \| \| 1.18635876 \| \| 1.44944472 \| \| 1.67201799 \| \| 2.05133445 \| \| 0.878262 \| \| 0.72924533 \| \| 0.27711185 \| \| 0.97470451 \| \| 2.4208286 \| \| 1.09582663 \| \| 0.00425359 \| \| 0.0012765 \| \| 0.11420203 \| \| 0.35339306 \| \| 0.41373068 \| \| 0.07828094 \| \| 0.86156754 \| \| 0.98109236 \| \| 1.75E-05 \| \| 0.15647751 \| \| 0.01095485 \| \| 0.05586027 \| \| 0.00077984 \| \| 0.04521327 \| \| 0.13597464 \| \| 0.09647244 \| \| 1.73104594 \| \| 1589.23599 \| \| 0.84285525 \| \| 1.79013724 \| \| 1.37348321 \| \| 0.93434123 \| \| 0.62832458 \| \| 0.99898122 \| \| 0.55320552 \| \| 0.47950454 \| \| 0.19366934 \| \| 0.43467224 \| \| 0.03113156 \| \| 0.04739338 \| \| 0.0237312 \| \| 0.0155282 \| | \| 0.09395611 \| \| --- \| \| 3.7999231 \| \| 0.06597648 \| \| 0.45794724 \| \| 0.01562571 \| \| 0.05301507 \| \| 0.14523858 \| \| 0.21095614 \| \| 0.68596711 \| \| 0.31703763 \| \| 1.64305631 \| \| 0.5704612 \| \| 0.46616103 \| \| 0.51639553 \| \| 0.35722325 \| \| 0.33781862 \| \| 18.4728149 \| \| 1.61332509 \| \| 0.5569797 \| \| 0.5992944 \| \| 0.77886054 \| \| 2.74979126 \| \| 1.25522378 \| \| 0.38834136 \| \| 2.35655484 \| \| 1.04460569 \| \| 0.03941222 \| \| 0.35305903 \| \| 0.43455981 \| \| 1.01711583 \| \| 1.85412199 \| \| 0.45618506 \| \| 10.5100278 \| \| 177.852286 \| \| 0.13881972 \| \| 1.59531153 \| \| 0.28256729 \| \| 3.17364489 \| \| 0.84084781 \| \| 0.75934911 \| \| 0.65582614 \| \| 1.99313181 \| \| 0.20766849 \| \| 0.30783718 \| \| 0.05624798 \| \| 0.30408731 \| \| 0.21787585 \| \| 0.2732741 \| \| 1.09341206 \| \| 2.34030954 \| \| 1.94390057 \| \| 1.49975506 \| \| 2.24071781 \| \| 1.38905082 \| \| 1.44533866 \| \| 1.87744155 \| \| 2.2621048 \| \| 3.22757722 \| \| 1.15140923 \| \| 0.8937568 \| \| 0.39816544 \| \| 1.16255308 \| \| 2.9488815 \| \| 1.41957049 \| \| 0.0072336 \| \| 0.00417936 \| \| 0.14927833 \| \| 0.43072507 \| \| 0.5926077 \| \| 0.09345129 \| \| 1.05245642 \| \| 1.26911685 \| \| 9.31E-05 \| \| 0.25562394 \| \| 0.01429841 \| \| 0.06783254 \| \| 0.00150951 \| \| 0.05407479 \| \| 0.16624717 \| \| 0.12470405 \| \| 2.34764107 \| \| 4778.90824 \| \| 1.09810355 \| \| 2.17506227 \| \| 1.9652848 \| \| 1.11106463 \| \| 0.7673075 \| \| 1.29845902 \| \| 0.74490336 \| \| 0.7584512 \| \| 0.25187074 \| \| 0.52994216 \| \| 0.04469108 \| \| 0.05663783 \| \| 0.02885361 \| \| 0.02008972 \| | \| 0.15934466 \| \| --- \| \| 9.62095074 \| \| 0.1179137 \| \| 0.6476327 \| \| 0.02815802 \| \| 0.08466487 \| \| 0.22719456 \| \| 0.34291428 \| \| 1.13961481 \| \| 0.80500506 \| \| 2.96764312 \| \| 0.81267433 \| \| 0.83686189 \| \| 0.8361687 \| \| 0.55664501 \| \| 0.5466966 \| \| 30.5283085 \| \| 4.12664524 \| \| 1.005087 \| \| 0.8460618 \| \| 1.41497525 \| \| 4.41046977 \| \| 1.95992016 \| \| 0.63099482 \| \| 3.94754919 \| \| 2.68417288 \| \| 0.07117456 \| \| 0.50156795 \| \| 0.78233913 \| \| 1.64283286 \| \| 2.88101739 \| \| 0.73983108 \| \| 17.6541245 \| \| 7373.64632 \| \| 0.24738481 \| \| 2.24984873 \| \| 0.50701734 \| \| 5.1118127 \| \| 1.30743329 \| \| 1.21984926 \| \| 1.10199267 \| \| 5.10533185 \| \| 0.3736792 \| \| 0.43599012 \| \| 0.10241752 \| \| 0.48820859 \| \| 0.34061438 \| \| 0.44196727 \| \| 1.83767913 \| \| 5.98848575 \| \| 3.46636544 \| \| 2.12962261 \| \| 4.06915258 \| \| 2.22279246 \| \| 2.24734687 \| \| 3.02520887 \| \| 3.77432918 \| \| 8.22249989 \| \| 2.06830366 \| \| 1.26167824 \| \| 0.72026435 \| \| 1.86597074 \| \| 4.61208191 \| \| 2.28037099 \| \| 0.01509547 \| \| 0.02358778 \| \| 0.26960011 \| \| 0.60785137 \| \| 1.07015416 \| \| 0.14986004 \| \| 1.64106877 \| \| 2.04689944 \| \| 0.00047041 \| \| 0.654367 \| \| 0.02573732 \| \| 0.09639295 \| \| 0.00336149 \| \| 0.08687394 \| \| 0.25930923 \| \| 0.20176177 \| \| 3.95746483 \| \| 28508.6678 \| \| 1.96895905 \| \| 3.11063888 \| \| 3.52612912 \| \| 1.79828435 \| \| 1.19510735 \| \| 2.10465015 \| \| 1.25693254 \| \| 1.95757983 \| \| 0.45245065 \| \| 0.75366848 \| \| 0.08068664 \| \| 0.09134575 \| \| 0.04497937 \| \| 0.03251795 \| |

DRE: Diet-responsive enteropathy. ARE: Antibiotic-responsive enteropathy. IRE: Immunosuppressant-responsive enteropathy. ‘Before’ corresponds to V1 in healthy dogs and active disease in CE dogs. ‘After’ corresponds to V2 in healthy dogs and remission in CE dogs. Top eleven of the most representative families. Other includes the rest of the families.
