# Supplementary material for: Hierarchical modelling of immunoglobulin coated bacteria in dogs with chronic enteropathy shows reduction in coating with disease remission but marked inter-individual and treatment-response variability
Source: PLoS One. 2021 Aug 19;16(8):e0255012. doi: 10.1371/journal.pone.0255012 (PMC8376084; doi:10.1371/journal.pone.0255012)
Supplement: S7 Table — (DOCX) [file pone.0255012.s013.docx]

**S7 Table. Estimates of the immunoglobulin G ratios and their credible intervals.**

| **Disease** | **Stage** | **Taxon** | **Q5** | **Q25** | **Q50** | **Q75** | **Q95** |
| --- | --- | --- | --- | --- | --- | --- | --- |
| \| IRE \| \| --- \| \| IRE \| \| DRE \| \| DRE \| \| ARE \| \| ARE \| \| Healthy \| \| Healthy \| \| IRE \| \| IRE \| \| DRE \| \| DRE \| \| ARE \| \| ARE \| \| Healthy \| \| Healthy \| \| IRE \| \| IRE \| \| DRE \| \| DRE \| \| ARE \| \| ARE \| \| Healthy \| \| Healthy \| \| IRE \| \| IRE \| \| DRE \| \| DRE \| \| ARE \| \| ARE \| \| Healthy \| \| Healthy \| \| IRE \| \| IRE \| \| DRE \| \| DRE \| \| ARE \| \| ARE \| \| Healthy \| \| Healthy \| \| IRE \| \| IRE \| \| DRE \| \| DRE \| \| ARE \| \| ARE \| \| Healthy \| \| Healthy \| \| IRE \| \| IRE \| \| DRE \| \| DRE \| \| ARE \| \| ARE \| \| Healthy \| \| Healthy \| \| IRE \| \| IRE \| \| DRE \| \| DRE \| \| ARE \| \| ARE \| \| Healthy \| \| Healthy \| \| IRE \| \| IRE \| \| DRE \| \| DRE \| \| ARE \| \| ARE \| \| Healthy \| \| Healthy \| \| IRE \| \| IRE \| \| DRE \| \| DRE \| \| ARE \| \| ARE \| \| Healthy \| \| Healthy \| \| IRE \| \| IRE \| \| DRE \| \| DRE \| \| ARE \| \| ARE \| \| Healthy \| \| Healthy \| \| IRE \| \| IRE \| \| DRE \| \| DRE \| \| ARE \| \| ARE \| \| Healthy \| \| Healthy \| | \| After \| \| --- \| \| Before \| \| After \| \| Before \| \| After \| \| Before \| \| After \| \| Before \| \| After \| \| Before \| \| After \| \| Before \| \| After \| \| Before \| \| After \| \| Before \| \| After \| \| Before \| \| After \| \| Before \| \| After \| \| Before \| \| After \| \| Before \| \| After \| \| Before \| \| After \| \| Before \| \| After \| \| Before \| \| After \| \| Before \| \| After \| \| Before \| \| After \| \| Before \| \| After \| \| Before \| \| After \| \| Before \| \| After \| \| Before \| \| After \| \| Before \| \| After \| \| Before \| \| After \| \| Before \| \| After \| \| Before \| \| After \| \| Before \| \| After \| \| Before \| \| After \| \| Before \| \| After \| \| Before \| \| After \| \| Before \| \| After \| \| Before \| \| After \| \| Before \| \| After \| \| Before \| \| After \| \| Before \| \| After \| \| Before \| \| After \| \| Before \| \| After \| \| Before \| \| After \| \| Before \| \| After \| \| Before \| \| After \| \| Before \| \| After \| \| Before \| \| After \| \| Before \| \| After \| \| Before \| \| After \| \| Before \| \| After \| \| Before \| \| After \| \| Before \| \| After \| \| Before \| \| After \| \| Before \| | \| *Bacteroidaceae* \| \| --- \| \| *Bacteroidaceae* \| \| *Bacteroidaceae* \| \| *Bacteroidaceae* \| \| *Bacteroidaceae* \| \| *Bacteroidaceae* \| \| *Bacteroidaceae* \| \| *Bacteroidaceae* \| \| *Clostridiaceae* \| \| *Clostridiaceae* \| \| *Clostridiaceae* \| \| *Clostridiaceae* \| \| *Clostridiaceae* \| \| *Clostridiaceae* \| \| *Clostridiaceae* \| \| *Clostridiaceae* \| \| *Coriobacteriaceae* \| \| *Coriobacteriaceae* \| \| *Coriobacteriaceae* \| \| *Coriobacteriaceae* \| \| *Coriobacteriaceae* \| \| *Coriobacteriaceae* \| \| *Coriobacteriaceae* \| \| *Coriobacteriaceae* \| \| *Enterobacteriaceae* \| \| *Enterobacteriaceae* \| \| *Enterobacteriaceae* \| \| *Enterobacteriaceae* \| \| *Enterobacteriaceae* \| \| *Enterobacteriaceae* \| \| *Enterobacteriaceae* \| \| *Enterobacteriaceae* \| \| *Erysipelotrichaceae* \| \| *Erysipelotrichaceae* \| \| *Erysipelotrichaceae* \| \| *Erysipelotrichaceae* \| \| *Erysipelotrichaceae* \| \| *Erysipelotrichaceae* \| \| *Erysipelotrichaceae* \| \| *Erysipelotrichaceae* \| \| *Fusobacteriaceae* \| \| *Fusobacteriaceae* \| \| *Fusobacteriaceae* \| \| *Fusobacteriaceae* \| \| *Fusobacteriaceae* \| \| *Fusobacteriaceae* \| \| *Fusobacteriaceae* \| \| *Fusobacteriaceae* \| \| *Lachnospiraceae* \| \| *Lachnospiraceae* \| \| *Lachnospiraceae* \| \| *Lachnospiraceae* \| \| *Lachnospiraceae* \| \| *Lachnospiraceae* \| \| *Lachnospiraceae* \| \| *Lachnospiraceae* \| \| Other \| \| Other \| \| Other \| \| Other \| \| Other \| \| Other \| \| Other \| \| Other \| \| *Paraprevotellaceae* \| \| *Paraprevotellaceae* \| \| *Paraprevotellaceae* \| \| *Paraprevotellaceae* \| \| *Paraprevotellaceae* \| \| *Paraprevotellaceae* \| \| *Paraprevotellaceae* \| \| *Paraprevotellaceae* \| \| *Prevotellaceae* \| \| *Prevotellaceae* \| \| *Prevotellaceae* \| \| *Prevotellaceae* \| \| *Prevotellaceae* \| \| *Prevotellaceae* \| \| *Prevotellaceae* \| \| *Prevotellaceae* \| \| *Ruminococcaceae* \| \| *Ruminococcaceae* \| \| *Ruminococcaceae* \| \| *Ruminococcaceae* \| \| *Ruminococcaceae* \| \| *Ruminococcaceae* \| \| *Ruminococcaceae* \| \| *Ruminococcaceae* \| \| *Veillonellaceae* \| \| *Veillonellaceae* \| \| *Veillonellaceae* \| \| *Veillonellaceae* \| \| *Veillonellaceae* \| \| *Veillonellaceae* \| \| *Veillonellaceae* \| \| *Veillonellaceae* \| | \| 0.04558632 \| \| --- \| \| 0.50783832 \| \| 0.04482964 \| \| 0.1792106 \| \| 0.02003663 \| \| 0.07579008 \| \| 0.09285974 \| \| 0.13844409 \| \| 0.30105379 \| \| 0.16247802 \| \| 0.22861034 \| \| 0.82452646 \| \| 0.10915345 \| \| 0.538568 \| \| 0.24306963 \| \| 0.17736945 \| \| 3.32E-05 \| \| 1.17102542 \| \| 0.09439909 \| \| 0.39645169 \| \| 0.0191706 \| \| 0.1795663 \| \| 0.16796412 \| \| 0.23057978 \| \| 0.25493686 \| \| 0.30008121 \| \| 0.78196828 \| \| 0.29098093 \| \| 0.10238625 \| \| 0.61329439 \| \| 0.13230237 \| \| 0.39450474 \| \| 0.11915058 \| \| 0.00018561 \| \| 0.23249586 \| \| 0.77455005 \| \| 0.25138532 \| \| 0.47971606 \| \| 0.34173801 \| \| 0.52305372 \| \| 0.1276518 \| \| 0.15444345 \| \| 0.19012442 \| \| 0.11679422 \| \| 0.03620761 \| \| 0.13446529 \| \| 0.13862889 \| \| 0.30520022 \| \| 0.66547428 \| \| 1.02649739 \| \| 1.05547831 \| \| 1.00656882 \| \| 1.05171895 \| \| 1.03953214 \| \| 1.04609231 \| \| 1.04237653 \| \| 1.38498992 \| \| 0.46848633 \| \| 0.57691571 \| \| 1.03433686 \| \| 0.11964529 \| \| 0.54320844 \| \| 1.00609765 \| \| 1.11430279 \| \| 0.13229584 \| \| 0.01566695 \| \| 0.08251501 \| \| 0.27094873 \| \| 0.3641151 \| \| 0.08800542 \| \| 0.38653693 \| \| 0.37107848 \| \| 3.00E-06 \| \| 0.51441901 \| \| 0.0114835 \| \| 0.08218065 \| \| 0.00671586 \| \| 0.07322162 \| \| 0.05510463 \| \| 0.06885245 \| \| 0.02804591 \| \| 615.982707 \| \| 0.4206065 \| \| 1.23763643 \| \| 0.5547589 \| \| 1.5944916 \| \| 0.71436165 \| \| 0.23836959 \| \| 0.248755 \| \| 0.27567037 \| \| 0.25634891 \| \| 0.29999039 \| \| 0.08270001 \| \| 0.15219013 \| \| 0.04734441 \| \| 0.05452314 \| | \| 0.0770639 \| \| --- \| \| 0.74715765 \| \| 0.05338475 \| \| 0.18791555 \| \| 0.02386361 \| \| 0.08554048 \| \| 0.10443426 \| \| 0.15426695 \| \| 0.51109793 \| \| 0.23955441 \| \| 0.27393852 \| \| 0.85831135 \| \| 0.12926075 \| \| 0.60970757 \| \| 0.27113259 \| \| 0.1972259 \| \| 0.00065324 \| \| 1.72736552 \| \| 0.11415845 \| \| 0.41776332 \| \| 0.02822412 \| \| 0.20269716 \| \| 0.19211548 \| \| 0.25837632 \| \| 0.43352387 \| \| 0.43970789 \| \| 0.9489006 \| \| 0.30161783 \| \| 0.12099747 \| \| 0.6931866 \| \| 0.1505357 \| \| 0.43871651 \| \| 0.20944248 \| \| 0.02449934 \| \| 0.27813923 \| \| 0.81257931 \| \| 0.29768865 \| \| 0.54271346 \| \| 0.38205546 \| \| 0.58270605 \| \| 0.21720897 \| \| 0.22530584 \| \| 0.22527426 \| \| 0.12287763 \| \| 0.04323809 \| \| 0.15181833 \| \| 0.15528316 \| \| 0.33899179 \| \| 1.13970155 \| \| 1.50526371 \| \| 1.25336281 \| \| 1.03580261 \| \| 1.24555412 \| \| 1.1739345 \| \| 1.16962809 \| \| 1.16067502 \| \| 2.35731932 \| \| 0.68360647 \| \| 0.68466804 \| \| 1.07221659 \| \| 0.14207071 \| \| 0.61236248 \| \| 1.12416568 \| \| 1.23945665 \| \| 0.22195813 \| \| 0.07137313 \| \| 0.09823579 \| \| 0.28069761 \| \| 0.4299122 \| \| 0.09939061 \| \| 0.43303373 \| \| 0.41364985 \| \| 3.57E-05 \| \| 0.77717653 \| \| 0.0136719 \| \| 0.08526017 \| \| 0.00902179 \| \| 0.08257384 \| \| 0.06165051 \| \| 0.07647831 \| \| 0.04984237 \| \| 1612.55332 \| \| 0.50148493 \| \| 1.28613352 \| \| 0.65695857 \| \| 1.79958442 \| \| 0.80181148 \| \| 0.2705948 \| \| 0.42778401 \| \| 0.40444765 \| \| 0.30483455 \| \| 0.31243396 \| \| 0.09943035 \| \| 0.17323199 \| \| 0.05316648 \| \| 0.06074416 \| | \| 0.126461 \| \| --- \| \| 1.02962659 \| \| 0.06787508 \| \| 0.19869073 \| \| 0.03182131 \| \| 0.10430516 \| \| 0.12267835 \| \| 0.17906879 \| \| 0.83871994 \| \| 0.32908013 \| \| 0.34911884 \| \| 0.90883835 \| \| 0.17246772 \| \| 0.74294691 \| \| 0.31887533 \| \| 0.22929254 \| \| 0.00367442 \| \| 2.42484612 \| \| 0.1462984 \| \| 0.44366875 \| \| 0.03956072 \| \| 0.24579159 \| \| 0.2256634 \| \| 0.2996267 \| \| 0.71679807 \| \| 0.60564484 \| \| 1.21843984 \| \| 0.31961665 \| \| 0.16130385 \| \| 0.84446516 \| \| 0.17611989 \| \| 0.50992197 \| \| 0.33447401 \| \| 0.54731761 \| \| 0.35313956 \| \| 0.86078253 \| \| 0.3971983 \| \| 0.66273041 \| \| 0.4476471 \| \| 0.67783505 \| \| 0.357579 \| \| 0.31155183 \| \| 0.28785733 \| \| 0.13014832 \| \| 0.05771167 \| \| 0.1843589 \| \| 0.18189547 \| \| 0.39408421 \| \| 1.88090579 \| \| 2.07751098 \| \| 1.60658268 \| \| 1.09642566 \| \| 1.66088107 \| \| 1.4303323 \| \| 1.37256965 \| \| 1.348889 \| \| 3.88470913 \| \| 0.94816671 \| \| 0.87468052 \| \| 1.13378564 \| \| 0.18903299 \| \| 0.7448392 \| \| 1.32015762 \| \| 1.44182658 \| \| 0.36398933 \| \| 0.20427138 \| \| 0.12503733 \| \| 0.29771724 \| \| 0.5751478 \| \| 0.12096963 \| \| 0.507656 \| \| 0.48098143 \| \| 0.00013147 \| \| 1.04343303 \| \| 0.01754207 \| \| 0.09052478 \| \| 0.01226722 \| \| 0.10041844 \| \| 0.07212624 \| \| 0.0887716 \| \| 0.08033773 \| \| 3722.42889 \| \| 0.63993928 \| \| 1.35967902 \| \| 0.87165965 \| \| 2.19681025 \| \| 0.94255546 \| \| 0.31471752 \| \| 0.70189802 \| \| 0.55983255 \| \| 0.39071398 \| \| 0.33076093 \| \| 0.13245412 \| \| 0.21110878 \| \| 0.06236278 \| \| 0.07046116 \| | \| 0.18636175 \| \| --- \| \| 1.3852983 \| \| 0.09345895 \| \| 0.2217296 \| \| 0.04901649 \| \| 0.13818943 \| \| 0.15684136 \| \| 0.2251671 \| \| 1.2246412 \| \| 0.44581383 \| \| 0.47895463 \| \| 1.01367716 \| \| 0.26607836 \| \| 0.98640039 \| \| 0.40656038 \| \| 0.28892041 \| \| 0.01356893 \| \| 3.32276582 \| \| 0.20098702 \| \| 0.49496085 \| \| 0.06116523 \| \| 0.32763101 \| \| 0.29045293 \| \| 0.3808563 \| \| 1.03282856 \| \| 0.8196818 \| \| 1.65276516 \| \| 0.35634818 \| \| 0.24784872 \| \| 1.11944124 \| \| 0.22522356 \| \| 0.64128239 \| \| 0.51146603 \| \| 10.1292378 \| \| 0.48673762 \| \| 0.95907655 \| \| 0.60844551 \| \| 0.87631801 \| \| 0.57163399 \| \| 0.85069803 \| \| 0.52109324 \| \| 0.42067567 \| \| 0.39686381 \| \| 0.14477845 \| \| 0.08831879 \| \| 0.2451756 \| \| 0.23279625 \| \| 0.49742034 \| \| 2.72387787 \| \| 2.80994057 \| \| 2.19835667 \| \| 1.22231104 \| \| 2.55021968 \| \| 1.89486682 \| \| 1.75101788 \| \| 1.6988931 \| \| 5.65107011 \| \| 1.28009399 \| \| 1.20317195 \| \| 1.26651283 \| \| 0.29060164 \| \| 0.99120284 \| \| 1.68826373 \| \| 1.8096531 \| \| 0.54583261 \| \| 0.54795208 \| \| 0.17279568 \| \| 0.33079127 \| \| 0.88346563 \| \| 0.1607466 \| \| 0.6491661 \| \| 0.60538654 \| \| 0.00038872 \| \| 1.44594123 \| \| 0.02410364 \| \| 0.1007198 \| \| 0.01895122 \| \| 0.13375448 \| \| 0.09216905 \| \| 0.11185896 \| \| 0.12454917 \| \| 10358.2873 \| \| 0.88097215 \| \| 1.51667591 \| \| 1.34229587 \| \| 2.90231145 \| \| 1.20513223 \| \| 0.39833931 \| \| 1.02873245 \| \| 0.7577824 \| \| 0.53416703 \| \| 0.36885881 \| \| 0.20396372 \| \| 0.28018411 \| \| 0.07974495 \| \| 0.08902366 \| | \| 0.38446073 \| \| --- \| \| 2.2808873 \| \| 0.15364854 \| \| 0.31034966 \| \| 0.10067872 \| \| 0.23250229 \| \| 0.24740369 \| \| 0.34561521 \| \| 2.51544902 \| \| 0.7267157 \| \| 0.79510966 \| \| 1.40644019 \| \| 0.54592156 \| \| 1.64263905 \| \| 0.65228069 \| \| 0.44553914 \| \| 0.06853558 \| \| 5.47300622 \| \| 0.33402162 \| \| 0.68429127 \| \| 0.13043405 \| \| 0.55150949 \| \| 0.46043335 \| \| 0.58934454 \| \| 2.11499034 \| \| 1.33052907 \| \| 2.74140863 \| \| 0.49625098 \| \| 0.50671781 \| \| 1.87549193 \| \| 0.3596303 \| \| 0.98442419 \| \| 1.06334446 \| \| 419.561988 \| \| 0.79428183 \| \| 1.34095476 \| \| 1.24127245 \| \| 1.47027651 \| \| 0.91270033 \| \| 1.30296836 \| \| 1.08143652 \| \| 0.68133086 \| \| 0.65570149 \| \| 0.20362851 \| \| 0.18151354 \| \| 0.41257063 \| \| 0.36855698 \| \| 0.75857073 \| \| 5.60367462 \| \| 4.54628165 \| \| 3.60825119 \| \| 1.70890931 \| \| 5.23297072 \| \| 3.17771587 \| \| 2.78982413 \| \| 2.59543209 \| \| 11.6994002 \| \| 2.05742947 \| \| 1.97993375 \| \| 1.76688578 \| \| 0.59792783 \| \| 1.67175001 \| \| 2.68235291 \| \| 2.77585886 \| \| 1.13467885 \| \| 2.02669625 \| \| 0.28389925 \| \| 0.46362678 \| \| 1.81729731 \| \| 0.27185211 \| \| 1.02688512 \| \| 0.92775269 \| \| 0.001542 \| \| 2.37599911 \| \| 0.03963813 \| \| 0.1410355 \| \| 0.03853214 \| \| 0.22672563 \| \| 0.14657903 \| \| 0.1713973 \| \| 0.26096426 \| \| 58500.7692 \| \| 1.44166591 \| \| 2.12385403 \| \| 2.77837857 \| \| 4.91816264 \| \| 1.92503861 \| \| 0.61317734 \| \| 2.09964865 \| \| 1.23064249 \| \| 0.88261352 \| \| 0.51553448 \| \| 0.42704332 \| \| 0.46892116 \| \| 0.12725545 \| \| 0.13656373 \| |

DRE: Diet-responsive enteropathy. ARE: Antibiotic-responsive enteropathy. IRE: Immunosuppressant-responsive enteropathy. ‘Before’ corresponds to V1 in healthy dogs and active disease in CE dogs. ‘After’ corresponds to V2 in healthy dogs and remission in CE dogs. Top eleven of the most representative families. Other includes the rest of the families.
