# Supplementary material for: Hierarchical modelling of immunoglobulin coated bacteria in dogs with chronic enteropathy shows reduction in coating with disease remission but marked inter-individual and treatment-response variability
Source: PLoS One. 2021 Aug 19;16(8):e0255012. doi: 10.1371/journal.pone.0255012 (PMC8376084; doi:10.1371/journal.pone.0255012)
Supplement: Attachment — Submitted filename: Response to reviewers.docx [file pone.0255012.s014.docx]

The authors thank the editor and reviewer for their comments. We hope that we have adequately addressed the suggestions made regarding the manuscript; and reached an appropriate standard for publication in your journal. The text has been tightened as suggested, and we note the statistical methodology used may be novel for many reviewers. We would welcome further review by a statistician should our responses not provide clarity.

1.A key limitation in this study is low animal numbers per group, resulting in very low power. Immune response and microbiota are highly variable among animals. This limitation should be listed first in that area of the Discussion section and an estimate on sufficient numbers in future studies should be estimated.

The limitation regarding the low number of animals enrolled in the study has been addressed in the discussion. Actual power is not directly a function of sample size but is instead related to the amount of information provided by the data with which effects are distinguished from noise. Power is a function of both the size of the effects of interest and the volume of information about them. In simple study designs the sample size (the number of subjects) is a reasonable indicator of the volume of information, but this is not the case in complex study designs, especially in the presence of repeated measures that greatly increase sensitivity by allowing the between-subject variance to be marginalized (moving between-subject variance from the ‘unexplained’ to the ‘explained’). Formally, the concept of power (β-error) is frequentist, which does not match the majority of the analyses presented in our case, but we will use ‘power’ generically to refer to the sensitivity of the analysis for estimates of interest given the available information [1].

In comments regarding power for a study that is already complete, the reader should engage with the precision of estimates of interest [2]. Intervals for those estimates demonstrate the sensitivity of the study (the informativeness about parameters) directly, and do so in the space of the results, not the design, and so naturally reflect the effect size. As we did, we think it appropriate to consider this review [3] regarding confidence intervals; the specific meaning of credible intervals is different, but the remaining concepts still apply. A small number of subjects is potentially an external validity limitation, but this has nothing to do with power. We have, however, removed that term to avoid confusion (line 865).

Regarding recommendations for sample sizes for future studies, we felt this was not the topic of the current paper and as it is a substantial methodological investment was outside the scope of this work; also, design analyses require information about the effect size of interest, and that is for determination by the researcher. It is important to note that our inclusion of estimated effects and their uncertainty, detailed description of statistical methodology, and provision of the data, would facilitate use by a future researcher in planning, whereas a null-hypothesis-significance-testing presentation would not.

2. There are 4 pages of statistical methods in the Methods section, but then there is little to no statistical data/p values given in the Results section, tables, and figures. The authors need to add these key details so that the reader can ascertain what was different (and can be discussed) and what was not.

An inherent limitation of microbiome trials is high dimensionality, in that there is inevitably a large volume of data and numerous statistical estimates. There is a natural trade-off between readability of the manuscript and the volume of literal numeric values presented in the results, so we have attempted to meet this challenge with a careful balance of visualization and direct numeric expression (supplementary figures added with the numerical values).

An important note regarding the reviewer’s request for *p*-values is that most of the data analysis in this work (models of flow cytometry and relative abundance data) is Bayesian, so *p*-values simply do not exist (these are a frequentist concept). More broadly, the reviewer should note that *p*-value driven inference has been deliberately rejected for inclusion in this work for multiple reasons. Most importantly is that presenting statistical results with emphasis on *p*-values obfuscates the actual quantities of interest which are effect size [4]. Additionally, there is substantial evidence that *p*-values are prone to misinterpretation and abuse [5-7]. For example, the expectation that *p*-values reflect statistical significance is a dangerous misinterpretation [8, 9].

More pragmatically, maximum likelihood methods (which may be compatible with *p*-values) are very challenging to implement in the longitudinal multinomial framework and require various adjustment for bias, and the available model structures are constrained by the capability of the estimating algorithms. In contrast, a Bayesian hierarchical model of the type presented here can be readily implemented without algorithmic limitations, and allows valid expression of uncertainty in the space of the results without complicated approximations. Popular strategies for microbiome data of simply splitting the data into many pieces and conducting numerous hypothesis significance tests are simply unsound [10], reflect nothing about effect size, and also demand engagement with type-1 error correction which is controversial and confusing [11].

We have oriented all of our presentations of uncertainty in both visual and tabulated form towards the estimated/predicted effects, which is more favourable for the reader.

Note line 449; the statement about ‘statistically significant’ shouldn’t be used for the above reasons, its presence was an oversight, and has been removed. We have also moved data to supplementary figures and reduced overall the length of the manuscript.

Specific comments:

1. Line 37, 132, and elsewhere: “found” and “seen” should be replaced with a more technical term

Terms have replaced by observed, detected, and identified.

2. Line 132-134: remained on same diet?

If the dogs went into remission (decrease by at least 75% of the clinical score), the same diet was continued. If there was only partial response, the dogs were reassessed two weeks later. If no further improvement was noted, another diet trial was pursued, before deciding if antibiotic treatment was required (i.e., no further response with the second diet trial). All dogs were still fed a therapeutic diet (this has been clarified in the relevant section).

3. Line 170: was pathologist blinded?

No. As the histological classification was not used to determine treatment groups, this was not thought necessary. Biopsies were performed simply to rule out non-inflammatory disease (i.e. neoplastic).

4. Line 262: 2.5 not 2,5

Corrected

5. Line 307: only 1,000 sequences used per sample? Why so low? Why go from 14,000 to 1,000 per sample?

We chose this value to minimise the loss of samples, for the positive population. In previous analysis, we performed the analysis at 8000 reads per sample. However, we lost 51 samples, so we decided to keep the analysis at 1000 reads per sample. Although the numerical values varied slightly, the same conclusions were reached.

6. Line 307: is there a citation for the Aitchison distance?

References added.

7. Line 448-449 and Table 1: why provide both? If sufficient data are provided in Figure 1, Table 2 can be deleted.

Table 2 has been provided as a supplementary table (S3 Table). The table provides important numerical information about the estimated effect sizes and their credible intervals.

8. Line 469-480: shorten and refer to the figure so there is no duplication.

Change has been done as suggested.

9. Line 485-486: delete sentence. This is anecdotal.

Sentenced deleted. However, we have kept the finding in our discussion as it is an important one. Due to the longitudinal nature of our study, we could observe that although remission is associated with decrease in the percentage of coating (as previously shown in cross-sectional studies in people), the opposite is not always true (not shown before).

10. Line 494-496: don’t give totals – it is not useful. Give the high-quality sequences used per sample.

Information deleted. High-quality sequences used were mentioned in the methods section.

11. Line 517 and elsewhere: what statistical value goes along with “decreased”? All differences noted need to be supported by p values. If significant p value does not exist, it is not different and should be stated as such (and not discussed).

*P* values provided for the Aitchison distance. However, for most of the cases, the degree of change is estimated, and there is an interval that summarizes the degree of uncertainty in the estimate. We have commented on *p*-values above and hope have addressed this sufficiently.

12. Line 520: why is it estimated? If Shannon index was calculated, why is it estimated?

In this case, though Shannon index is strictly speaking an inferred quantity, we have treated it as input data as the response in a statistical model. The table then summarizes the average Shannon index by condition, along with the precision of estimation (S4 table). The purpose of the table is to summarize the statistical inference, not to describe data. This is described in the methodology section.

13. Line 521 and 524 and elsewhere: why are insignificant words capitalized?

Changes have been made as suggested here and elsewhere.

14. Lines 538-541: there is still a lot of clustering within dog in ill animals too. Unless statistics support this statement, it should be revised.

Although some pattern of clustering is evident in sick dogs, samples were more dispersed, except in some DRE dogs, where all samples clustered together over the course of the study. In ARE and IRE, although some clustering pattern can be observed, not all samples from the same dog clustered in close proximity (The closer the distance between the two samples, the more similar is their bacterial community). Paragraph has been rephrased. It is worth pointing out that a *p*-value does not exist because there is no coherent test hypothesis associated with ‘degree of clustering’, and this is exclusively a visual assessment.

15. Lines 558-565: are there statistics to support these statements?

Supplementary tables have been provided with the estimates (S6 and S7 tables).

16. Lines 626-627: do statistics support this?

Please refer to figure 3 and S4 table.

17. Line 627: do not refer to figures or tables in Discussion. Those should only be in the Results section.

Changes have been made as suggested.

18. Line 634: revise “made in dogs” so it is an appropriate statement.

Corrected

19. Line 649: trespass is misspelled

Corrected

20. Lines 662-665: likely due to low power, correct? This needs to be highlighted early on.

We do not think there is value in talking about power once the study has already been done; see linked references above. We did, however, mention that these findings have been consistent with previous reports as specified in line 516 (refs 32, 33).

21. Lines 683-690: did statistics support this? If not remove or revise.

We decided to remove to reduce the length of the manuscript.

22. Line 703: which families? Should list specifics here so results can be compared to literature.

Changes have been made as suggested.

23. Lines 733-738, 753-754, and elsewhere: these need to be clearly shown in Results section with appropriate statistics if it is going to be discussed here.

Please refer to figure 5 and S6 and S7 tables.

24. Lines 844-847: this is the key issue and should be listed first.

Please refer to responses to comment 1.

25. Lines 877-883: this is not a conclusion, but future study ideas. Remove and add more information from the current study.

Changes have been made as suggested.

26. Figure 3: estimated? Why not just Shannon index?

The figure is presenting the model-predicted (i.e. estimated) average Shannon index by condition. This is an inferred quantity, not an observation (though strictly speaking the Shannon index is itself an estimate, not an observation). Figure caption has been rephrased to reflect the results given by the model.

27. Supplementary Table 3: predicted – not actual?

The objective of the table (now S4 Table) is to represent the statistical inference of the effects of predictors on the Shannon index and not to provide a summarize of the observations. Table caption has been adjusted to be more explicit.

# **References**

1.	Morey, R., *Why you shouldn’t say “this study is underpowered”*. 2019: Available from: <https://towardsdatascience.com/why-you-shouldnt-say-this-study-is-underpowered-627f002ddf35>.

2.	Hoenig, J.M. and D.M. Heisey, *The Abuse of Power.* The American Statistician, 2001. **55**(1): p. 19-24.

3.	Colegrave, N. and G.D. Ruxton, *Confidence intervals are a more useful complement to nonsignificant tests than are power calculations.* Behavioral Ecology, 2003. **14**(3): p. 446-447.

4.	Gardner, M.J. and D.G. Altman, *Confidence intervals rather than P values: estimation rather than hypothesis testing.* British Medical Journal (Clinical research ed.), 1986. **292**(6522): p. 746-750.

5.	Colquhoun, D., *The reproducibility of research and the misinterpretation of p-values.* R Soc Open Sci, 2017. **4**(12): p. 171085.

6.	McShane, B.B., et al., *Abandon Statistical Significance.* The American Statistician, 2019. **73**(sup1): p. 235-245.

7.	Cristea, I.A. and J.P.A. Ioannidis, *P values in display items are ubiquitous and almost invariably significant: A survey of top science journals.* PLoS One, 2018. **13**(5): p. e0197440.

8.	Goodman, S., *A Dirty Dozen: Twelve P-Value Misconceptions.* Seminars in Hematology, 2008. **45**(3): p. 135-140.

9.	Greenland, S., et al., *Statistical tests, P values, confidence intervals, and power: a guide to misinterpretations.* European Journal of Epidemiology, 2016. **31**(4): p. 337-350.

10.	Harrison, J.G., et al., *Dirichlet-multinomial modelling outperforms alternatives for analysis of microbiome and other ecological count data.* Molecular Ecology Resources, 2020. **20**(2): p. 481-497.

11.	Streiner, D.L., *Best (but oft-forgotten) practices: the multiple problems of multiplicity—whether and how to correct for many statistical tests.* The American Journal of Clinical Nutrition, 2015. **102**(4): p. 721-728.
